# Angiopoietin-like protein 2 mediates vasculopathy-driven fibrogenesis in a mouse model of systemic sclerosis

**DOI:** 10.1172/JCI177123

**Published:** 2025-06-20

**Authors:** Dyuti Saha, Ravi Kiran Annadorai, Sujaya Thannimangalath, Neha P. Shroff, Sunny Kataria, Binita Dam, Abhik Dutta, Akshay Hegde, Ankita Hiwale, Venkatesh Ravula, Shagnik Saha, Lekshmi Minikumari Rahulan, Neha Nigam, Neha Singh, Vikas Agarwal, Praveen K. Vemula, Colin Jamora

**Affiliations:** 1 IFOM-inStem Joint Research Laboratory, Institute for Stem Cell Science and Regenerative Medicine, Bangalore, India.; 2 Department of Biology, Manipal Academy of Higher Education, Manipal, India.; 3 Department of Life Sciences, Shiv Nadar Institution of Eminence, Delhi-NCR, India.; 4 School of Chemical and Biotechnology, Shanmugha Arts, Science, Technology and Research Academy, Thanjavur, Tamil Nadu, India.; 5 Laboratory of Chemical Biology and Translational Research, Institute for Stem Cell Science and Regenerative Medicine (DBT-inStem), Bangalore, India.; 6 Center of Excellence in Epigenetics, Department of Life Sciences, Shiv Nadar Institution of Eminence, Delhi-NCR, India.; 7 Department of Clinical Immunology and Rheumatology and; 8 Department of Pathology, Sanjay Gandhi Postgraduate Institute of Medical Sciences, Lucknow, India.

**Keywords:** Cell biology, Dermatology, Vascular biology, Endothelial cells, Fibrosis, Skin

## Abstract

Vasculopathy is a common hallmark of various fibrotic disorders, including systemic sclerosis (SSc), yet its underlying etiology and contribution to fibrogenesis remain ill defined. In SSc, the vasculopathy typically precedes the onset of fibrosis, and we observed that this phenomenon is recapitulated in the *Snail* transgenic mouse model of SSc. The vascular anomalies manifest as deformed vessels, endothelial cell dysfunction, and vascular leakage. Our investigation into the underlying mechanism of this phenotype revealed that angiopoietin-like protein 2 (ANGPTL2), secreted by the *Snail* transgenic keratinocytes, is a principal driver of fibrotic vasculopathy. In endothelial cells, ANGPTL2 upregulates profibrotic genes, downregulates various junctional proteins, and prompts the acquisition of mesenchymal characteristics. Inhibiting endothelial cell junctional instability and, consequently, vascular leakage with a synthetic analog of the microbial metabolite Urolithin A (UAS03) effectively mitigated the vasculopathy and inhibited fibrogenesis. Thus, ANGPTL2 is a promising early biomarker of the disease, and inhibiting the vasculopathy-inducing effects of this protein with agents such as UAS03 presents an appealing therapeutic avenue to reduce disease severity. These insights hold the potential to revolutionize the approach to treatment of fibrotic diseases by targeting vascular defects.

## Abstract 2

Angiopoietin-like protein 2 induces vasculopathy that drives cutaneous fibrogenesis similar to that found in systemic sclerosis

## Graphical abstract

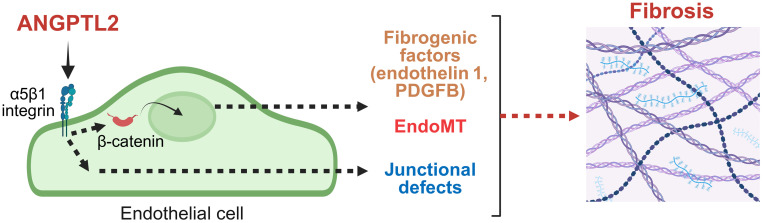

## Introduction

Fibrosis is a pathological condition wherein deposition of extracellular matrix (ECM) components by activated fibroblasts leads to tissue stiffness and loss of function. Fibrotic diseases lead to approximately 40% of deaths worldwide (1); however, the lack of effective treatments emphasizes the need for a thorough molecular understanding of fibrogenesis. In this context, the role of soluble factors such as TGF-β (2) and immune cells and inflammation (3) has been extensively studied for their contribution toward fibrosis development. However, targeting these pathways in clinical trials has had limited success (4), suggesting the existence of compensatory/overlapping signaling cascades that require elucidation to facilitate effective therapy development.

Fibrotic skin diseases range from localized fibrotic lesions on the skin (seen in localized scleroderma) to conditions with widespread involvement of multiple organs in systemic sclerosis (SSc) (5, 6). Like many other fibrotic tissues, defects in blood vessels in the skin are a prominent hallmark of SSc (7). Interestingly, patient data indicate that these vasculopathies manifest quite early in the disease progression (8), suggesting their potential role in promoting fibrogenesis. Examination of patient samples from SSc has revealed that the disease is marked by early endothelial damage, followed by the accumulation of immune cell infiltrates near the affected vasculature and eventually foci of ECM deposits by activated fibroblasts (9, 10). The early manifestation of vascular defects in the course of fibrogenesis suggests that pathways and factors involved in mediating vascular abnormalities could potentially be used both as early biomarkers of the disease as well as therapeutic targets. While there have been a few mouse models developed to understand the mechanistic basis of SSc progression, most do not recapitulate all the facets of the disease condition (11, 12). For example, the genetic mouse model of SSc, *Tsk2/+*, exhibits dermal fibrosis and inflammation but shows an absence of vascular defects (13, 14), whereas the *Tsk1/+* mouse exhibits abnormal vascular tone but lacks a robust inflammatory response (15, 16). By contrast, chemically induced models such as the bleomycin-induced fibrotic model (17, 18) faithfully recapitulate the dermal fibrosis and inflammation associated with SSc while only partially recapitulating the vasculature defects. Therefore, there was a need for a mouse model system that recapitulates a larger spectrum of SSc characteristics to elucidate the molecular mechanism of SSc vasculopathy.

We previously developed a transgenic (tg) mouse model mimicking SSc skin by ectopically expressing the transcription factor Snail in the basal layer of keratinocytes in mouse skin. Snail has been reported to be upregulated in various fibrotic conditions (19–22), including SSc (23). *Snail*-tg mouse skin recapitulates various histological and molecular features of SSc, such as increased dermal thickness, ECM deposition, and increased inflammation. Similar to the progression of SSc, fibrosis in the *Snail*-tg mouse is long-lasting and originates in the skin and progresses to the involvement of internal organs (23). Although defects related to blood vessels, such as Raynaud’s phenomenon, were observed in the *Snail*-tg skin (23), it remains to be determined to what extent *Snail*-tg skin recapitulates SSc vasculopathy. The presence of SSc-like vascular defects in *Snail*-tg skin positions this mouse model as a platform to elucidate the underlying mechanisms of SSc vasculopathy and the contribution of vascular defects to fibrogenesis.

## Results

### SSc-associated vasculopathy is recapitulated in adult Snail-tg skin.

**Figure 1:**
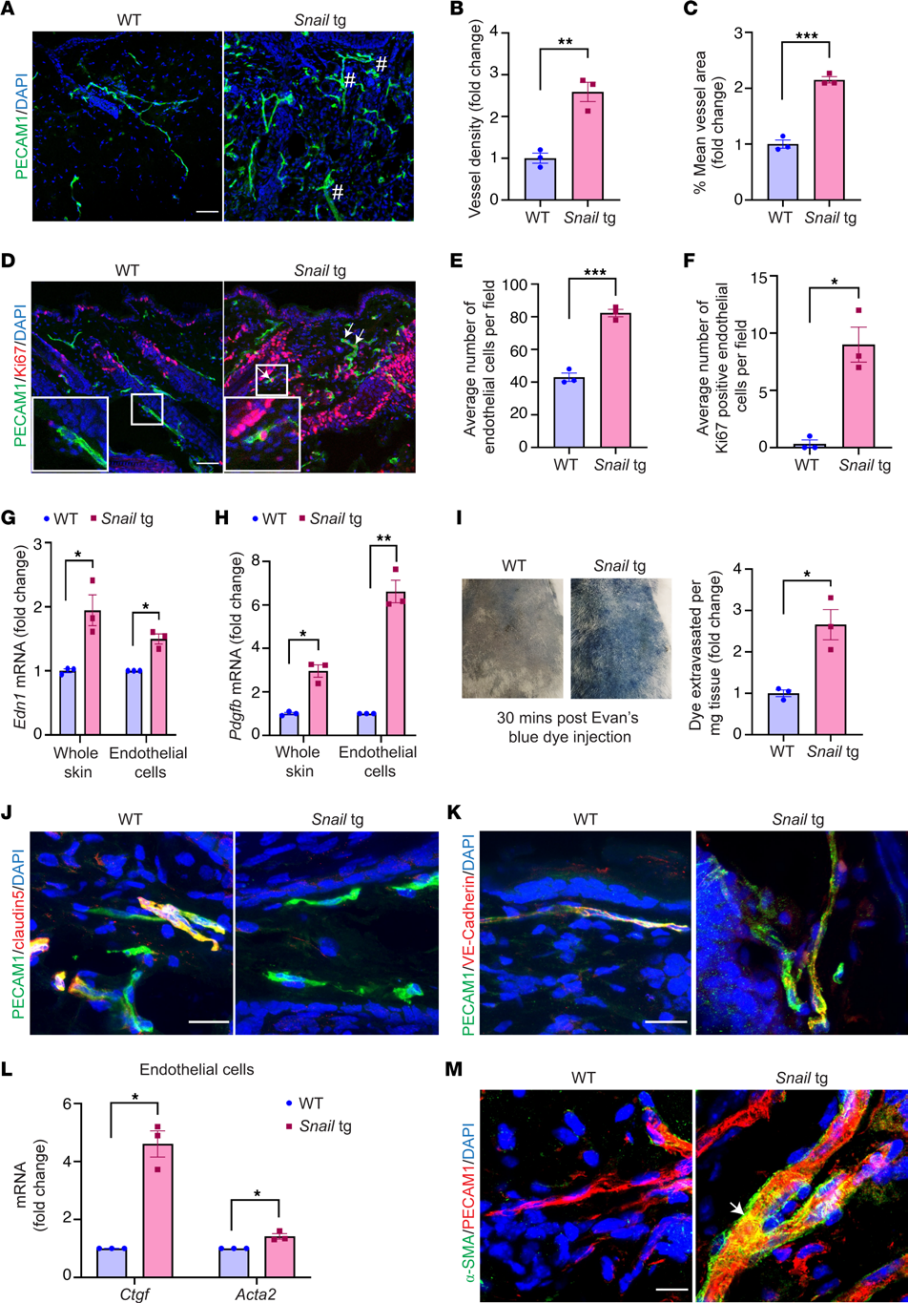
Adult *Snail*-tg skin recapitulates vasculature abnormalities observed in SSc. (**A**) WT and *Snail*-tg skin stained for PECAM1 (green). Dilated regions of vessels are denoted with a pound symbol. Quantification of (**B**) vessel density and (**C**) mean vessel area in WT and *Snail*-tg skin. (**D**) WT and *Snail*-tg skin stained for PECAM1 (green) and proliferation marker Ki67 (red). Arrows mark PECAM1^+^/Ki67^+^ cells. Insets show magnified endothelial cells. Number of (**E**) endothelial cells and (**F**) PECAM1^+^/Ki67^+^ cells in WT and *Snail*-tg skin. qPCR analysis of (**G**) *Edn1* mRNA and (**H**) *Pdgfb* mRNA in whole skin and isolated dermal endothelial cells in WT and *Snail*-tg mice. (**I**) Evans blue dye leakage assay and quantification. WT and *Snail*-tg skin stained for (**J**) Claudin5 (red)/PECAM1 (green) and (**K**) VE-Cadherin (red)/PECAM1(green). (**L**) qPCR analysis of myofibroblast markers (*Ctgf* and *Acta2*) in isolated dermal endothelial cells from WT and *Snail*-tg mice skin. (**M**) WT and *Snail*-tg skin stained for α-SMA (green) and PECAM1 (red). Arrow marks α-SMA^+^/PECAM1^+^ vascular structure. Nuclei are marked in blue. Scale bars: 50 μm (**A** and **D**), 20 μm (**J** and **K**), 10 μm (**M**). Data are shown as mean ± SEM; *P* values were calculated using unpaired Welch’s *t* test for whole skin data and paired Student’s *t* test for endothelial cell data. *P < 0.05, **P < 0.01, ***P < 0.001. All experiments are *n* = 3 biological replicates. Three fields of interest (FOIs) were analyzed per biological replicate for quantification of images.

We previously reported that the *Snail*-tg mouse recapitulates many of the diagnostic features of SSc (23–25). We thus investigated whether *Snail*-tg mouse skin could recapitulate the various aspects of vasculopathy associated with SSc. We undertook a morphometric analysis of the cutaneous vasculopathy in 2-month-old adult *Snail*-tg mice with the markers used to profile for human (26) and mouse (18, 27) fibrotic skin. Staining of the back skin of an adult *Snail*-tg mouse with the endothelial cell marker PECAM1 (platelet endothelial cell adhesion molecule 1) revealed that *Snail*-tg skin vessels had increased morphological distortions in the form of altered vascular structures compared with its WT counterpart (Figure 1A). Upon performing morphometric analysis, we observed a significant increase in vascular density (Figure 1B) and percentage of mean vessel area (Figure 1C) in *Snail*-tg skin compared with the WT skin. Distorted vasculature is often an early driver of pathological neovascularization with hyperproliferation of endothelial cells (28). Consistent with this, we observed a 2-fold increase in the total number of endothelial cells and approximately 9-fold increase in the number of Ki67-positive endothelial cells in the *Snail*-tg skin vasculature compared with WT skin (Figure 1, D–F).

We next analyzed gene expression changes related to SSc vasculopathy. Endothelin 1 (*Edn1*), a marker of endothelial cell damage and dysfunction found in SSc skin (29, 30), was increased in the *Snail*-tg skin (Figure 1G). Another important factor that is upregulated in SSc patients is platelet-derived growth factor B (PDGFB), which is constitutively secreted from endothelial cells and affects both perivascular cells as well as activate surrounding fibroblasts (31). *Pdgfb* is likewise upregulated in the whole skin and isolated dermal endothelial cells of *Snail*-tg mouse compared with WT (Figure 1H).

To investigate whether the blood vessels in *Snail*-tg skin are functionally compromised, we examined the vascular integrity with the Evans blue dye leakage assay. We observed that the *Snail*-tg skin had increased blue color, indicating vascular leakage. Upon quantification, we found that *Snail*-tg skin had a nearly 3-fold increased dye extravasation into tissue from the vasculature, indicating the perturbation of vessel integrity (Figure 1I). We hypothesized that disruption of endothelial cell–cell adhesion underlies the increased vascular permeability in the *Snail*-tg skin and analyzed the status of the tight junction protein claudin5. In *Snail*-tg skin, the vessels exhibited a reduction in claudin5 levels (Figure 1J), and upon quantification, we observed a significant decrease in PECAM1 and claudin5 double-positive vascular structures (Supplemental Figure 1A; supplemental material available online with this article; https://doi.org/10.1172/JCI177123DS1). Similarly, upon analysis of the adhesion junction protein VE-Cadherin (vascular endothelial cadherin), PECAM1 and VE-Cadherin double-positive vascular structures were also significantly reduced in *Snail*-tg skin (Figure 1K and Supplemental Figure 1B). In line with this, we found that isolated endothelial cells from *Snail*-tg skin exhibited a decrease in gene expression of various junction proteins, such as claudin5 (*Cldn5*), VE-Cadherin (*Cdh5*), ZO1 (*Tjp1*), occludin (*Ocln*), and JAM3 (*Jam3*) (Supplemental Figure 1C). The loss of endothelial barrier stability has been hypothesized to prime endothelial cells to adopt a mesenchymal phenotype (32, 33). A partial or complete acquisition of mesenchymal characteristics can cause endothelial cells to adopt features of myofibroblasts in SSc skin (28, 34). We observed upregulated mRNA expression of the myofibroblast genes *Ctgf* (connective tissue growth factor) and *Acta2* (α-smooth muscle actin [α-SMA]) in isolated endothelial cells from *Snail*-tg skin (Figure 1L). Also, the *Snail*-tg skin exhibited a significant increase in α-SMA/PECAM1 double-positive vessels when compared with WT skin (Figure 1M and Supplemental Figure 1D).

Overall, *Snail*-tg skin reproduces key vascular abnormalities observed in SSc, providing a valuable platform to profile the developmental progression of the vasculopathy and its molecular underpinnings.

### Developmental analysis of Snail-tg skin revealed that vasculopathy defects occur early in fibrogenesis.

**Figure 2:**
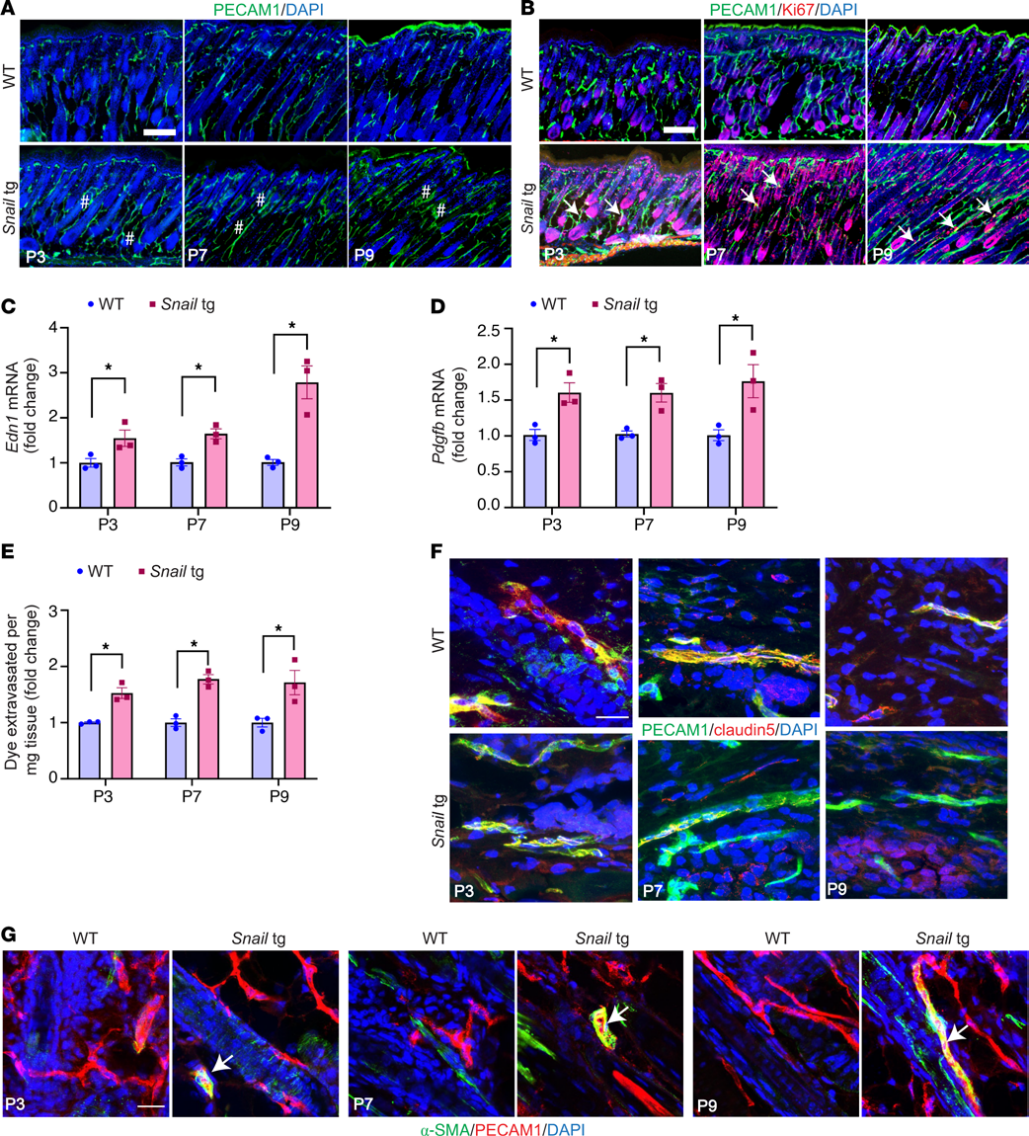
Vasculopathy phenotype occurs early in fibrogenesis. The skin from P3, P7, and P9 WT and *Snail*-tg mice was stained for (**A**) PECAM1 (green) and for (**B**) PECAM1 (green) and Ki67 (red). Dilated regions are marked by a pound symbol, and arrows mark PECAM1^+^/Ki67^+^ cells. qPCR analysis of (**C**) *Edn1* mRNA and (**D**) *Pdgfb* mRNA in whole skin in WT and *Snail*-tg mice. (**E**) Quantification of Evans blue dye leakage assay. WT and *Snail*-tg skin stained for (**F**) claudin5 (red) and PECAM1 (green) and for (**G**) α-SMA (green) and PECAM1 (red). Arrow marks α-SMA/PECAM1 double-positive vascular structures. Nuclei are marked in blue. Scale bars: 100 μm (**A** and **B**), 20 μm (**F** and **G**). Data are shown as mean ± SEM; *P* values were calculated using unpaired Welch’s *t* test. *P < 0.05. All experiments are *n* = 3 biological replicates.

Since vasculopathy is an early phenomenon in SSc disease progression (7), we hypothesized that it may have an active role in promoting fibrogenesis. To interrogate whether *Snail*-tg skin also exhibits early vasculopathy that precedes the thickening of the dermis, we performed a developmental analysis of the vasculopathy phenotype. Previously, we observed increased dermal thickening (a readout for fibrosis) as early as postnatal day 9 in *Snail*-tg skin, which increased progressively to adulthood (23). We therefore analyzed the vascular defects via PECAM1 staining at P3, P7, and P9 and observed an increase in vessel density and percentage of mean vessel area as early as P3 in *Snail*-tg skin (Figure 2A and Supplemental Figure 2, A and B). Moreover, an increase in the overall number of endothelial cells as well as Ki67/PECAM1 double-positive cells at P3 indicated the hyperproliferation of endothelial cells (Figure 2B and Supplemental Figure 2, C and D). Likewise, markers of endothelial damage, such as *Edn1* (Figure 2C) and *Pdgfb* (Figure 2D), were upregulated initially from P3, suggesting that signaling pathways mediating vascular defects are initiated early in disease development in *Snail*-tg skin. We further tested the vascular integrity at these stages by performing an Evans blue dye leakage assay. As early as P3 *Snail*-tg skin exhibited increased vascular permeability (Figure 2E). In line with this, we detected disruptions in claudin5 starting at P3, where it appeared in a punctate pattern on the surface of certain vascular structures (Figure 2F). Additionally, the number of vascular structures coexpressing claudin5 and PECAM1 significantly decreased (Supplemental Figure 2E). Moreover, some α-SMA/PECAM1 double-positive vessels were first observed at P3 and became more prominent in P7 *Snail*-tg skin (Figure 2G and Supplemental Figure 2F). Notably, this suggests that changes related to endothelial–mesenchymal transition (endoMT) in these vessels are linked to junctional destabilization.

Overall, our findings suggest that vasculopathy emerges in *Snail*-tg skin before marked dermal thickening develops, mirroring the early vascular defects seen in SSc patient skin.

### Angiopoietin-like protein 2 secreted by Snail-tg keratinocytes is necessary to induce dermal vasculopathy.

**Figure 3:**
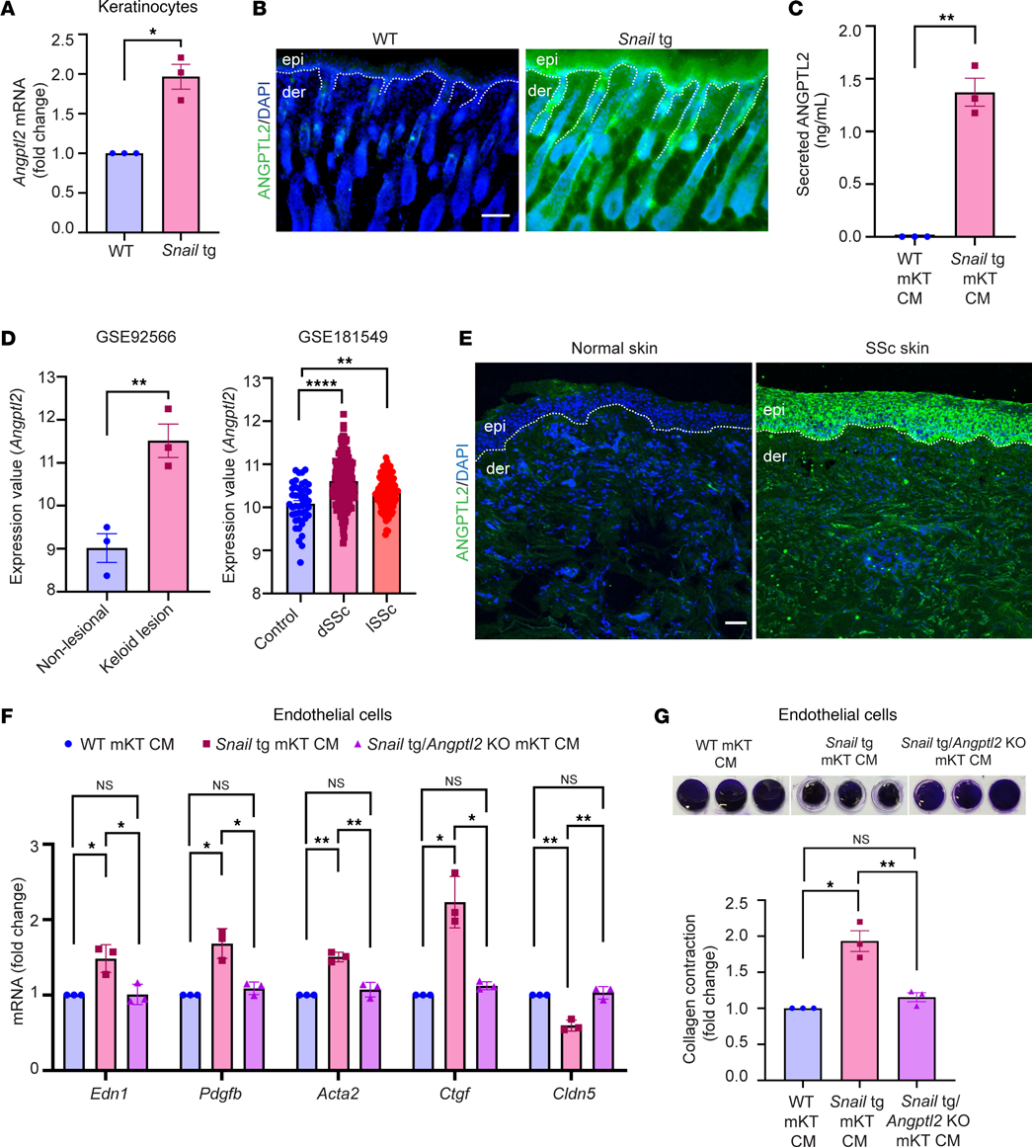
ANGPTL2 is necessary for fibrotic vasculopathy. (**A**) qPCR of *Angptl2* mRNA in WT and *Snail*-tg mouse keratinocytes (mKT). (**B**) Secreted ANGPTL2 (in green) in WT and *Snail*-tg skin at P3. The dotted line denotes the basement membrane separating the epidermis (epi) from the underlying dermis (der). (**C**) ELISA for secreted ANGPTL2 in WT and *Snail*-tg mKT conditioned media (CM). (**D**) Expression of *Angptl2* in keloid lesion skin (*n* = 3) compared with nonlesion skin (*n* = 3) (from GSE92566) and in healthy (*n* = 44) compared with affected forearm skin from diffuse (*n* = 180) and localized SSc (*n* = 115) patients (from GSE181549). The expression values are from the GEO2R algorithm’s output. (**E**) Secreted ANGPTL2 (in green) in normal and SSc skin. Representative of *n* = 5 SSc samples and normal skin controls. (**F**) qPCR of vasculopathy and endoMT-related genes in SVEC4-10 endothelial cells treated with WT, *Snail*-tg, and *Snail*-tg/*Angptl2*-KO mKT conditioned media. (**G**) Quantification of collagen contraction assay in SVEC4-10 endothelial cells treated with WT, *Snail*-tg, and *Snail*-tg/*Angptl2*-KO mKT conditioned media. Nuclei are marked in blue. Scale bars: 50 μm. Data are shown as mean ± SEM; *P* values were calculated using paired Student’s *t* test (**A**), unpaired Welch’s *t* test (**D**), and 1-way ANOVA followed by Tukey’s post hoc analysis (**F** and **G**). *P < 0.05, **P < 0.01, ****P < 0.0001, NS > 0.05. All experiments except (**D** and **E**) are *n* = 3 biological replicates.

Previously, we reported that extracellular factors such as Mindin (23) and Plasminogen activator inhibitor-1 (PAI1) (24) are secreted from *Snail*-tg keratinocytes and elicit fibrotic responses by activating dermal fibroblasts. We thus investigated whether the secretome of *Snail*-tg keratinocytes can elicit the vascular defects found in the transgenic mouse. Conditioned media from *Snail*-tg keratinocytes induced vascular thickening and increased density in WT skin explants that recapitulated the vasculopathy found in the transgenic skin (Supplemental Figure 3, A and B). The next question was the identity of the vasculopathy-inducing factors in the secretome of *Snail*-tg keratinocytes. Though Mindin or PAI1 play critical roles in fibrogenesis in *Snail*-tg skin, they are not required for vasculopathy (Supplemental Figure 3, C–F). Another factor that is highly expressed (Figure 3A) and secreted (Figure 3, B and C) primarily from the epidermal keratinocytes of *Snail*-tg skin at P3 is angiopoietin-like protein 2 (ANGPTL2) (Supplemental Figure 4A). Bioinformatic analysis of *Angptl2* gene expression in published datasets of keloid (35) and SSc (36) patients and fibrotic diseases of other human organs, such as the lung (37), kidney (38), and liver (39), revealed that *Angptl2* is upregulated in diseased skin compared with healthy skin controls (Figure 3D and Supplemental Figure 4, B–D). In addition, ANGPTL2 has been reported to be involved in pathological angiogenesis in diseases such as cancer (40). Therefore, we hypothesized that ANGPTL2 might also play a role in fibrotic vasculopathy in SSc skin. We found that ANGPTL2 is secreted from SSc patient skin, suggesting a possible contribution to disease manifestation (Figure 3E).

To test the role of ANGPTL2 in SSc vasculopathy, we generated a *Angptl2*-KO mouse in the *Snail*-tg background (Supplemental Figure 4E). We first assessed whether the presence of ANGPTL2 was required for vasculopathy-related characteristics in cultured endothelial cells. Conditioned media containing secreted proteins from *Snail*-tg keratinocytes upregulated vasculopathy-related genes such as *Edn1* and *Pdfgb*, while promoting an endoMT phenotype, as assessed by a collagen contraction assay. Notably, these vasculopathy-related effects were abrogated when using conditioned media from *Snail-tg/Angptl2*-KO keratinocytes (Figure 3, F and G).

**Figure 4:**
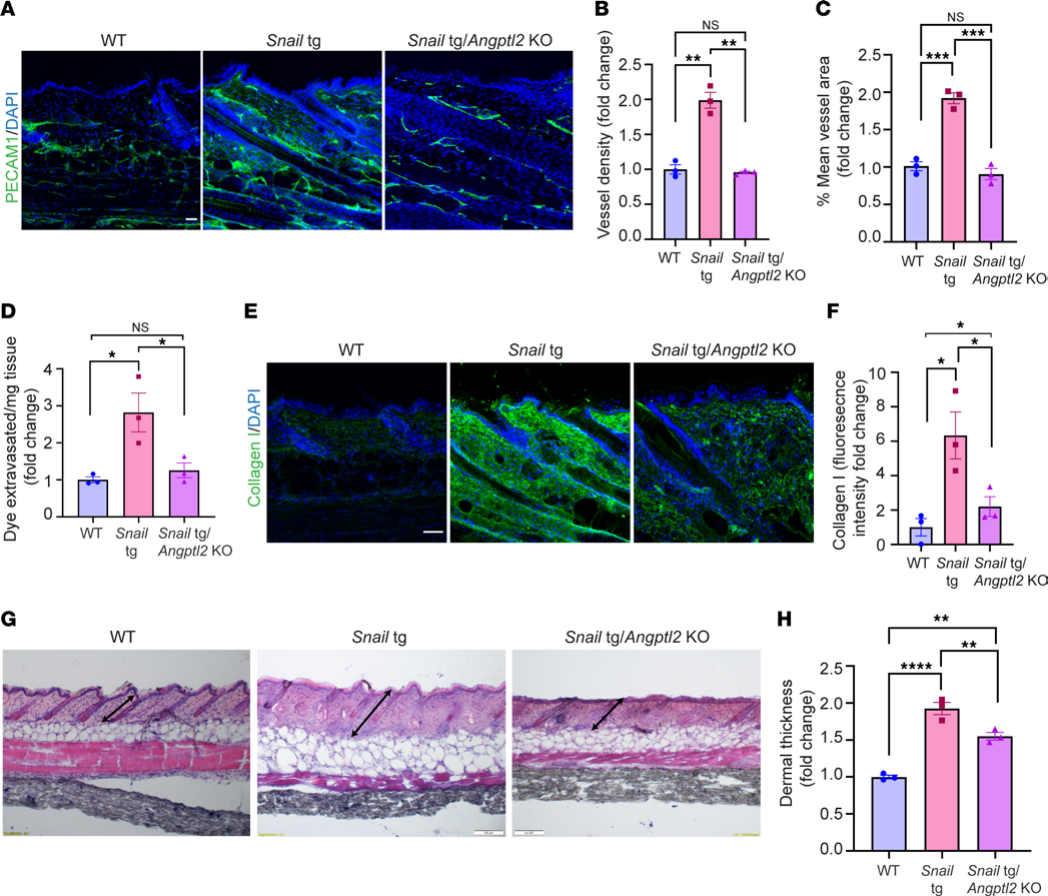
ANGPTL2 is required for development of vasculopathy and fibrosis in *Snail*-tg skin. (**A**) PECAM1 (green) staining, (**B**) quantification of vessel density, (**C**) quantification of vessel area, (**D**) quantification of Evans blue dye leakage assay, (**E**) collagen I staining (green) and (**F**) quantification, and (**G**) H&E staining and (**H**) quantification of dermal thickness in WT, *Snail*-tg, and *Snail*-tg/*Angptl2*-KO mouse at P60. Scale bars: 50 μm (**A** and **E**), 100 μm (**G**). Nuclei are marked in blue. Data are shown as mean ± SEM: *P* values were calculated using 1-way ANOVA followed by Tukey’s post hoc analysis for multiple group comparisons. *P < 0.05, **P < 0.01, ***P < 0.001, ****P < 0.0001, NS > 0.05. All experiments are *n* = 3 biological replicates. Three FOIs were analyzed per biological replicate for quantification of images.

Further, we assessed the necessity of ANGPTL2 in vasculopathy and fibrogenesis in the *Snail*-tg adult mouse in vivo. We found that the vascular density (Figure 4, A–C) and vascular permeability (Figure 4D) in the *Snail*-tg*/Angptl2*-KO mouse was restored to WT levels. Furthermore, the increased collagen I levels (Figure 4, E and F) and dermal thickness (Figure 4, G and H) (readout of fibrosis) in the *Snail*-tg skin was significantly reduced in the absence of ANGPTL2. Overall, these data indicate that ANGPTL2 is an important factor that drives the vasculopathy and fibrotic phenotypes in *Snail*-tg skin.

### ANGPTL2 is sufficient to drive various features of dermal vasculopathy.

**Figure 5:**
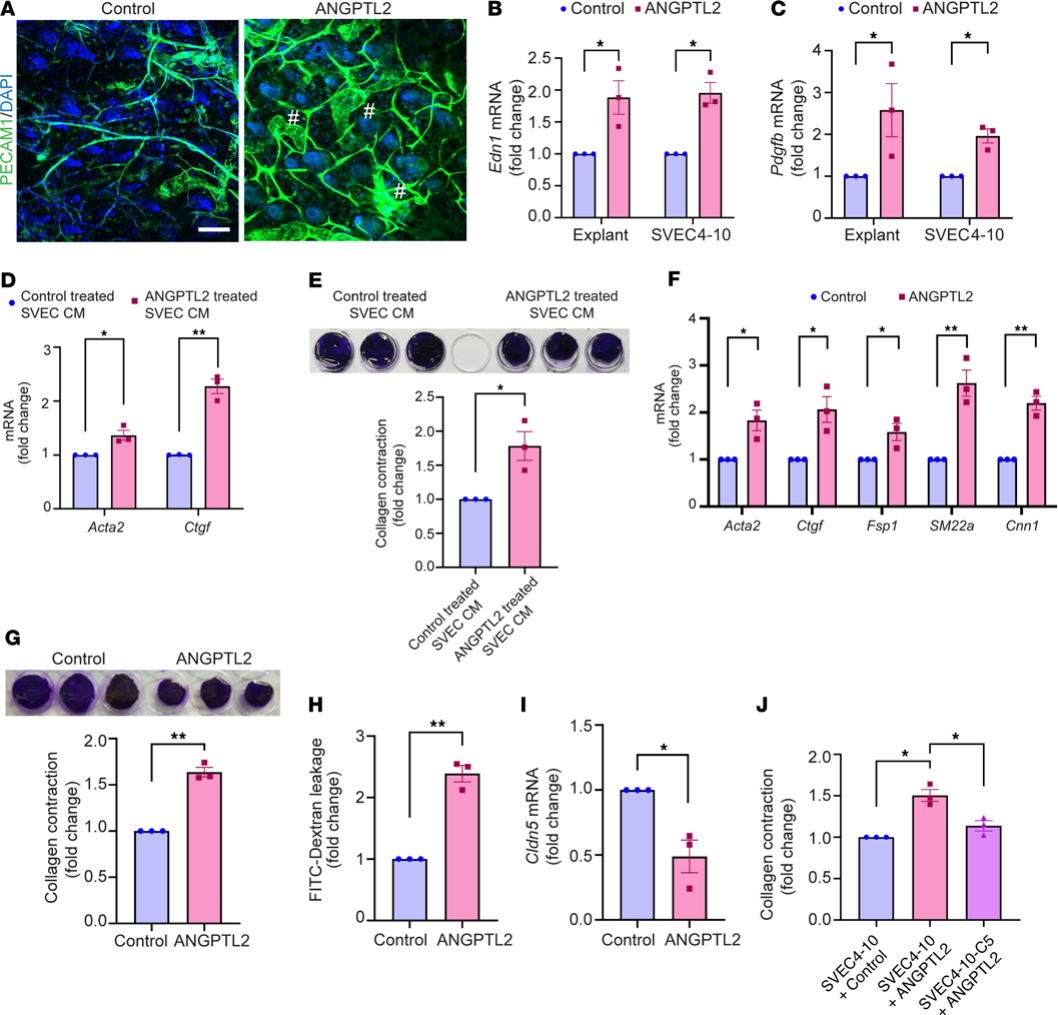
ANGPTL2 is sufficient to drive vasculopathy. (**A**) PECAM1 (green) staining in control and ANGPTL2-treated skin explants. The pound symbols denote dilated regions. Nuclei are marked in blue. qPCR analysis of mRNA levels of (**B**) *Edn1* and (**C**) *Pdgfb* in control and ANGPTL2-treated skin explants and SVEC4-10 cells. (**D**) qPCR analysis of mRNA levels of myofibroblast markers (*Acta2* and *Ctgf*). (**E**) Collagen contraction assay (left panel) and quantification (right panel) using dermal fibroblasts treated with conditioned media from control and ANGPTL2-treated SVEC4-10 cells. (**F**) qPCR analysis of mRNA levels of myofibroblasts markers (*Acta2*, *Ctgf*, *Fsp1*, *SM22a*, and *Cnn1*) in control and ANGPTL2-treated SVEC4-10. (**G**) Collagen contraction assay (left panel) and quantification (right panel) for control and ANGPTL2-treated SVEC4-10. (**H**) Quantification of in vitro vascular permeability assay. (**I**) qPCR analysis of mRNA levels of *Cldn5* in control and ANGPTL2-treated SVEC4-10. (**J**) Quantification of collagen contraction assay in SVEC4-10 + control, SVEC4-10 + ANGPTL2, and claudin5-overexpressed SVEC4-10 (SVEC4-10-C5) + ANGPTL2. Scale bar: 50 μm. Data are shown as mean ± SEM; *P* values were calculated using paired Student’s *t* test (**B**–**I**) and 1-way ANOVA followed by Tukey’s post hoc analysis (**J**). *P < 0.05, **P < 0.01. All experiments are *n* = 3 biological replicates.

We investigated the extent to which ANGPTL2 is sufficient to drive the vasculopathy observed in the skin of the *Snail*-tg mouse. Using an ex vivo explant assay, we found that ANGPTL2 can promote the dilation and swelling of vessels (Figure 5A), which recapitulates the changes in the vasculature in *Snail*-tg skin. ANGPTL2 also caused significant upregulation in mRNA levels of factors involved in SSc vasculopathy, such as *Edn1* (Figure 5B) and *Pdgfb* (Figure 5C), in skin explants and the mouse endothelial cell line SVEC4-10, an endothelial cell line derived by SV40 transformation of endothelial cells from mouse axillary lymph node vessels. In line with this, secreted factors from ANGPTL2-treated endothelial cells could drive upregulation of the myofibroblast genes *Acta2* and *Ctgf* (Figure 5D) as well as induce increased contraction (Figure 5E) in dermal fibroblasts. However, ANGPTL2 did not have a direct effect on endothelial cell proliferation in vitro (Supplemental Figure 5, A and B).

Endothelial cells isolated from *Snail*-tg skin exhibited upregulation of myofibroblast markers, underlying our hypothesis that ANGPTL2 could play a role in promoting endoMT. Levels of myofibroblast markers (*Acta2*, *Ctgf*, *Fsp1* [Fibroblast specific protein 1], *SM22a* [transgelin], and *Cnn1* [calponin 1]) were upregulated in SVEC4-10 cells upon ANGPTL2 treatment (Figure 5F and Supplemental Figure 5, C and D). Further, ANGPTL2 mediated a change in actin distribution (Supplemental Figure 5E), which is consistent with endothelial cells undergoing endoMT (41, 42). These observations recapitulate colocalization of fibroblast and endothelial markers (43) found in human SSc. We further examined whether these changes in the characteristics of endothelial cells resulted in alterations in vasculature functions. ANGPTL2 imparted contractile behavior in endothelial cells in collagen matrices (Figure 5G) and induced vascular leakage in a sheet of SVEC4-10 cells in vitro (Figure 5H).

Vascular dysfunction is often a consequence of perturbed tight junctions due to the loss of claudin5 expression (44). Consistent with this, quantitative PCR (qPCR) analysis revealed that mRNA levels of the tight junction protein claudin5 were significantly downregulated in SVEC4-10 cells treated with ANGPTL2 (Figure 5I). Moreover, the distribution of claudin5 and another junctional protein (VE-Cadherin) was perturbed in treated endothelial cells (Supplemental Figure 5, F and G). Considering the important role of claudin5 in maintaining vascular integrity (44, 45), we further tested if the acquisition of mesenchymal behaviors was likewise dependent on claudin5. To counteract the ANGPTL2-mediated loss of claudin5, we generated a stable SVEC4-10 cell line with constitutive claudin5 overexpression (SVEC4-10-C5). SVEC 4-10-C5 cells were refractory to increased contractility in collagen matrices upon ANGPTL2 treatment when compared with the parental SVEC4-10 cells (Figure 5J).

**Figure 6:**
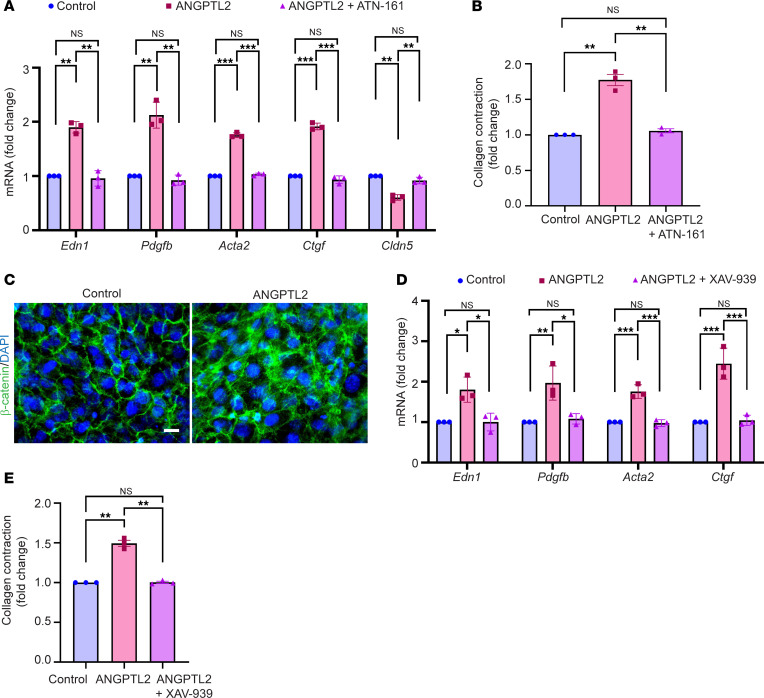
ANGPTL2-driven vasculopathy is mediated by the integrin α_5_β_1_/β-catenin pathway. (**A**) qPCR analysis of mRNA levels of vasculopathy and endoMT-related genes and (**B**) collagen contraction assay quantification in SVEC4-10 cells treated with control, ANGPTL2, and ANGPTL2 + ATN-161. (**C**) Staining for β-catenin (green) in control or ANGPTL2-treated SVEC4-10 cells. (**D**) qPCR analysis of mRNA levels of vasculopathy and endoMT-related genes and (**E**) collagen contraction assay quantification in SVEC4-10 cells treated with control, ANGPTL2, and ANGPTL2 + XAV-939. Scale bar: 10 μm. Data are shown as mean ± SEM; *P* values were calculated using 1-way ANOVA followed by Tukey’s post hoc analysis for multiple group comparisons. *P < 0.05, **P < 0.01, ***P < 0.001, NS > 0.05. All experiments are *n* = 3 biological replicates.

We then investigated the signaling pathway activated by ANGPTL2 in endothelial cells. The integrin α_5_β_1_ has been reported as a receptor for ANGPTL2 in other contexts (46); therefore, we tested if ANGPTL2’s effect in our system was dependent on this receptor. We found that blocking integrin α_5_β_1_ using the specific inhibitor, ATN-161, a non-RGD based integrin binding peptide targeting α_5_β_1_ abrogates the effects of ANGPTL2 (Figure 6, A and B). Previous reports indicated that β-catenin (a signaling component closely associated with integrin α_5_β_1_ and junctional proteins) has roles in mediating endoMT (47). Interestingly, endothelial cells treated with ANGPTL2 exhibit a change in β-catenin localization (Figure 6C). Inhibiting β-catenin using XAV-939 inhibitor (a small molecule specifically targeting and inhibiting tankyrase 1 involved in the Wnt signaling pathway) blocked the increased gene expression of *Edn1* and *Pdgfb* (profibrotic factors) as well as *Acta2* and *Ctgf* (markers of activated fibroblasts) (Figure 6D). Moreover, inhibition of β-catenin abrogated the ANGPTL2-induced increase in the contractile behavior of the cells (Figure 6E). Altogether, these data reveal the crucial role of β-catenin as a downstream mediator of ANGPTL2’s effect on the endoMT and the production of profibrotic factors.

### ANGPTL2-mediated vasculopathy is abrogated by a synthetic analog of Urolithin A.

**Figure 7:**
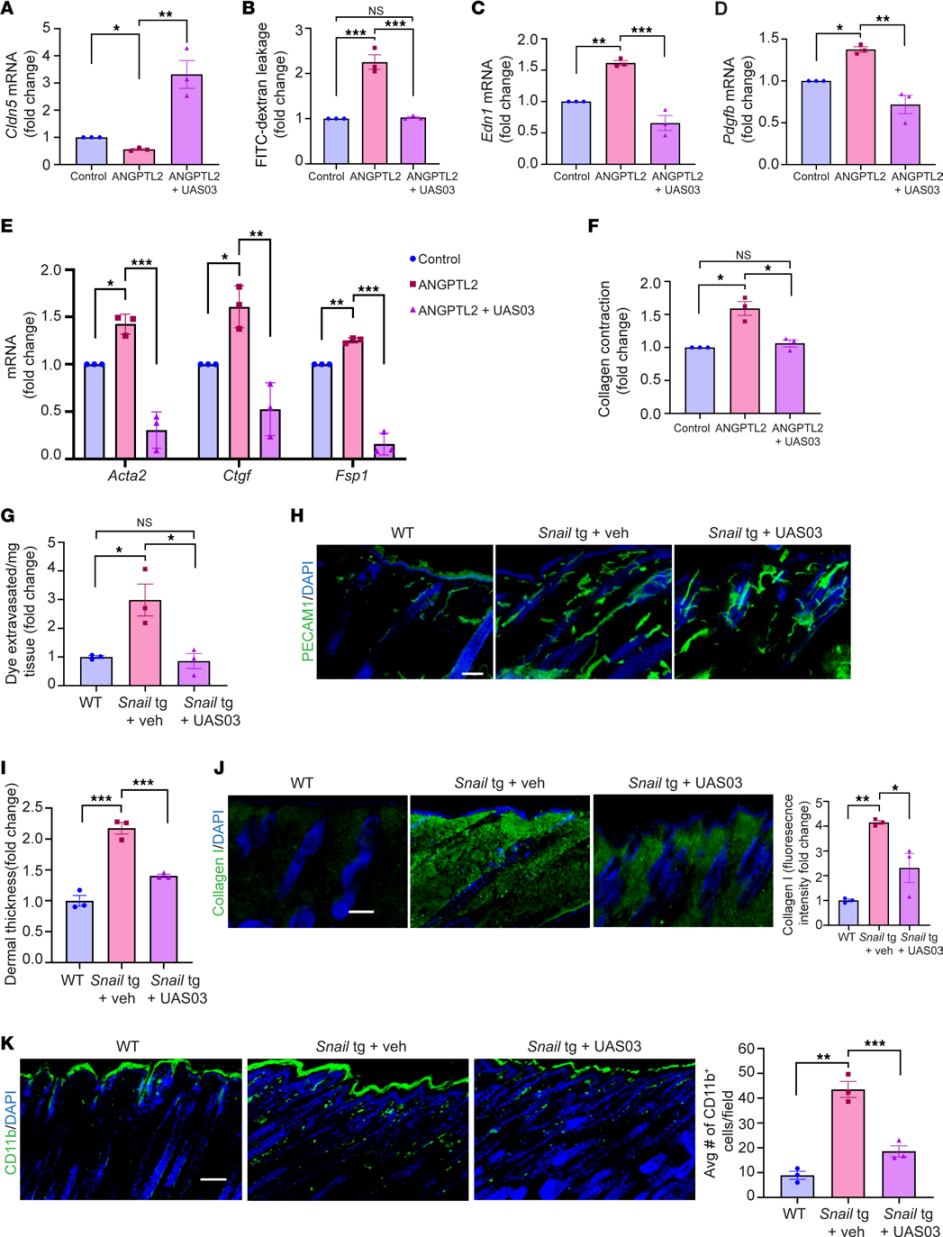
UAS03 abrogates effects of ANGPTL2 on endothelial cells and counteracts fibrosis. SVEC4-10 cells were treated with control, ANGPTL2, or ANGPTL2 + UAS03 and processed for qPCR of *Cldn5* mRNA (**A**), in vitro vascular permeability (**B**), qPCR of *Edn1* mRNA (**C**), qPCR of *Pdgfb* mRNA (**D**), expression of myofibroblast markers (*Acta2*, *Ctgf*, and *Fsp1*) (**E**), and collagen contraction activity (**F**). (**G**) Quantification of Evans blue dye leakage assay. (**H**) Staining for PECAM1 (green) and nuclei (DAPI) at P60 in WT, *Snail*-tg + vehicle control (veh), and *Snail*-tg + UAS03 injected mice. (**I**) Quantification of dermal thickness at P60 in WT, *Snail*-tg + veh, and *Snail*-tg + UAS03. (**J**) Staining for collagen I (green) and quantification at P60 in WT, *Snail-tg* + veh, and *Snail*-tg + UAS03. (**K**) Staining for CD11b^+^ cells (green) and nuclei (blue) and quantification at P9 in WT, *Snail*-tg + veh, and *Snail*-tg + UAS03. Scale bars: 100 μm (**H** and **J**), 50 μm (**K**). Data are shown as mean ± SEM; *P* values were calculated using paired Student’s *t* test for (**A**–**F**) and 1-way ANOVA followed by Tukey’s post hoc analysis for multiple group comparisons for (**G** and **I**–**K**). *P < 0.05, **P < 0.01, ***P < 0.001, NS > 0.05. All experiments are *n* = 3 biological replicates. Three FOIs were analyzed per biological replicate for quantification of images.

We investigated whether upregulation of tight junction proteins such as claudin5 through chemical modalities would be an effective mechanism to inhibit ANGPTL2-driven vasculopathy. We used a synthetic analog of the gut metabolite Urolithin A (UAS03), which was previously shown to restore the disrupted epithelial barrier by upregulating tight junction proteins in a mouse model of inflammatory bowel disease (48). We tested whether this function is conserved for endothelial cell junctions and found that UAS03 treatment counteracts the decrease of claudin5 mRNA induced by ANGPTL2 in SVEC4-10 cells (Figure 7A). In line with this effect on gene expression, UAS03 could also block the ANGPTL2-induced vascular leakage through an endothelial sheet in vitro (Figure 7B). We further interrogated if UAS03 could counteract the other vasculopathy defects mediated by ANGPTL2. Increased mRNA levels of *Edn1* (Figure 7C) and *Pdgfb* (Figure 7D) induced by ANGPTL2 treatment were restored to control levels in the presence of UAS03. We also observed that UAS03 treatment decreased mRNA levels of the endoMT markers *Acta2*, *Ctgf*, and *Fsp1* induced by ANGPTL2 (Figure 7E). Consistent with the gene expression changes, UAS03 also blocked ANGPTL2-mediated increased contractility of endothelial cells in collagen matrices (Figure 7F). In sum, our data reveal that utilization of UAS03 is an effective method for mitigating the vasculopathy induced by ANGPTL2 in vitro.

We next interrogated whether UAS03 is effective in reducing the fibrotic phenotype in vivo. Intraperitoneal injections of UAS03 in mice from neonates (P3) to adulthood (P60) reduced the vascular leakage in the adult *Snail*-tg animals to WT levels (Figure 7G). Moreover, both vascular density and mean vessel area were significantly reduced upon injection of UAS03 in *Snail*-tg skin (Figure 7H and Supplemental Figure 6, A and B). We tested if the protective effects of UAS03 on the vasculature likewise impact fibrogenesis in the *Snail*-tg mice. We observed a significant decrease in dermal thickness (Figure 7I) and collagen I protein levels (Figure 7J) in UAS03-injected *Snail*-tg mice compared with vehicle control at P60.

Inflammation is a hallmark of fibrosis and is facilitated by the recruitment of immune cells into tissue through leaky vessels. Among these immune cells, macrophages are well known to be increased in many fibrotic conditions. We observed that the approximately 4-fold increase of CD11b^+^ cells (macrophages) in *Snail*-tg skin was substantially decreased in UAS03-injected transgenic mice (Figure 7K). We thus assessed if the UAS03-mediated decrease in the dermal fibrosis in *Snail*-tg skin is secondary to its effect on macrophage reduction. Interestingly, depletion of macrophage number with the chemical clodronate (Supplemental Figure 7, A and B) was not sufficient to reduce the increased dermal thickness (Supplemental Figure 7C) and collagen I protein levels (Supplemental Figure 7, D and E) in *Snail*-tg mice skin compared with vehicle control.

### UAS03 enhances tight junction while inhibiting pro-angiogenic/fibrogenic gene expression.

**Figure 8:**
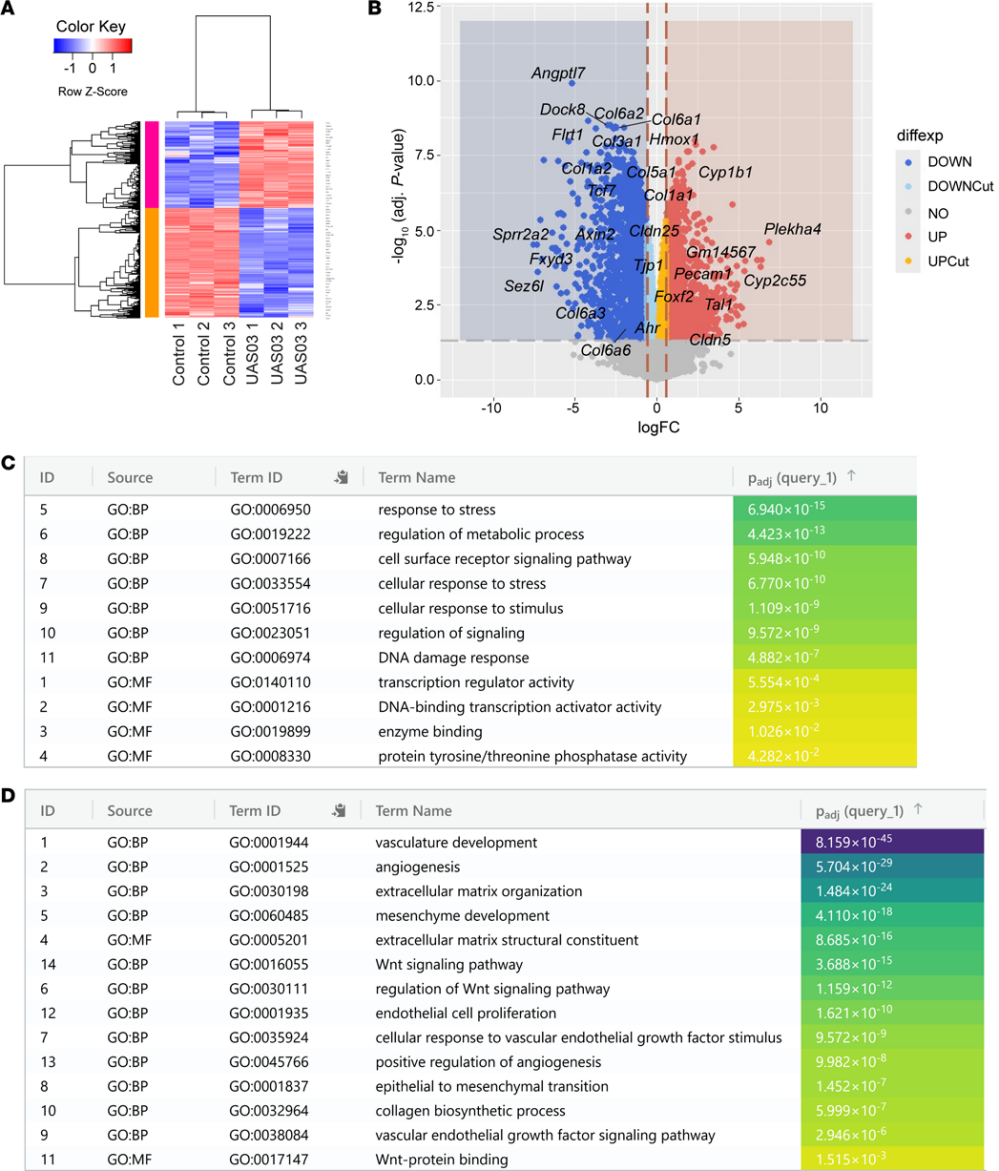
RNA-Seq analysis of UAS03-treated endothelial cells. SVEC4-10 cells were treated with control or 50 μM UAS03, total RNA was isolated, and RNA-Seq was performed as described in Methods. (**A**) Heat map of differentially expressed genes generated using the heatmap.2 function in the gplots package. Genes with *q* value of less than 0.05 and fold change (FC) greater than1.5 or less than –1.5 were clustered using the hclust function using Pearson’s method for genes and Spearman’s method for samples. (**B**) Volcano plot of differentially expressed genes created using ggplot. Downregulated genes are represented as blue dots (*q* < 0.05, FC < –1.5) and light blue dots (*q* < 0.05, FC < 0, FC > –1.5). Upregulated genes are represented as red dots (*q* < 0.05, FC > 1.5) and gold dots (*q* < 0.05, FC > 0, FC < –1.5). The following genes are highlighted: top 3 significant differentially expressed genes, top 3 upregulated and downregulated genes with maximum fold change, and genes of interest based on the in vivo observations with UAS03 treatment. GO term enrichment analysis from the list of significantly upregulated (**C**) and downregulated (**D**) genes using gProfiler2. Terms of interest from “molecular function” and “biological pathway” categories are depicted based on the in vivo observations with UAS03 treatment. *n* = 3 biological replicates.

To examine the mechanism of action of UAS03 on the vasculature in *Snail*-tg skin, we performed RNA sequencing (RNA-Seq) analysis of SVEC4-10 endothelial cell line treated with this synthetic metabolite. Based on an adjusted *P* value (*q* value) of 0.05, 7,554 genes were differentially expressed (Supplemental Table 1). Furthermore, based on a fold change cutoff of 1.5, 1,658 genes were significantly upregulated and 2,093 genes were significantly downregulated (Figure 8, A and B). Among the top genes based on significance and fold change, genes such as *Angptl7* and *Fxyd3*, which are implicated in pro-angiogenic effects, were downregulated upon treatment with UAS03 (49, 50). By contrast, among the top upregulated genes was *Cyp2c55*, which has been reported to have roles in physiological functions in endothelial cells such as maintenance of vascular tone (51). This unbiased approach suggests that UAS03 has an overall role in maintaining the homeostatic state of endothelial cells.

Given the previous report from the Vemula group (48) and our data (Figure 7A), we focused on the expression of endothelial junction proteins in the RNA-Seq data. In addition to *Cldn5*, *Cldn25* was upregulated in UAS03-treated cells (Figure 8B). Furthermore, the expression of the junction protein *Pecam1*, which has been previously reported to promote the barrier integrity of endothelial cells (52), was also upregulated in UAS03-treated cells. In addition, we also observed upregulation of the tight junction protein *Tjp1* by UAS03 in the dataset; however, it was below the 1.5-fold cutoff used for our analysis (absolute fold change: 1.26). Interestingly, the adhesion junction protein VE-Cadherin (*Cdh5*) did not exhibit a statistically significant change in the RNA-Seq dataset, but we observed an increased expression through qPCR analysis (Supplemental Figure 8). Altogether, these data indicate that UAS03 promotes endothelial junction stabilization through upregulation of junction components. This provides a mechanistic framework for the ability of UAS03 to inhibit vascular leakage in *Snail*-tg skin and thereby impede fibrogenesis (Figure 7).

Gene Ontology (GO) term enrichment analysis using gProfiler2 (Supplemental Table 2) revealed upregulation of genes involved in “response to stress” and “DNA damage response” (Figure 8C). For instance, genes such as *Hmox1* and *Cyp1b1*, which have protective roles against oxidative damage, were among the upregulated genes (Figure 8B). This is consistent with reports of UAS03’s protective role against oxidative stress in gut epithelial cells (48) and suggests this function may likewise be conserved in endothelial cells.

Our data demonstrate that UAS03 treatment in endothelial cells and *Snail*-tg mice reduced vasculopathy and fibrosis (Figure 7). In line with this, processes such as “angiogenesis,” “positive regulation of angiogenesis,” “endothelial cell proliferation,” “epithelial to mesenchymal transition,” and “mesenchyme development” were within the downregulated category (Figure 8D). This implies that UAS03 can provide a protective effect against ANGPTL2 by downregulating genes that would otherwise promote pathological angiogenesis and acquisition of mesenchymal phenotypes in endothelial cells. In addition, we found that terms such as “extracellular matrix structural constituents” and “collagen biosynthetic process” were also within the downregulated category. In line with this, various collagen genes such as *Col1a1* were downregulated by UAS03 (Figure 8B). This indicates that UAS03 suppresses profibrotic gene expression in endothelial cells, potentially contributing to the decreased fibrosis observed in treated *Snail*-tg mice.

Our data suggest that various vasculopathy-related gene expression changes mediated by ANGPTL2 are driven through β-catenin (Figure 6, C–E). Notably, terms such as “Wnt signaling pathway” and “Wnt protein binding” were in the downregulated category, suggesting that UAS03 is capable of suppressing β-catenin signaling pathways that fuel fibrosis.

Altogether, these observations support the notion that vascular defects have an important contribution to fibrogenesis in the *Snail*-tg mouse. Moreover, these findings position the synthetic metabolite UAS03 as a potential therapeutic strategy against vasculopathy in fibrotic skin conditions such as SSc and thereby substantially reduce tissue fibrosis.

## Discussion

**Figure 9:**
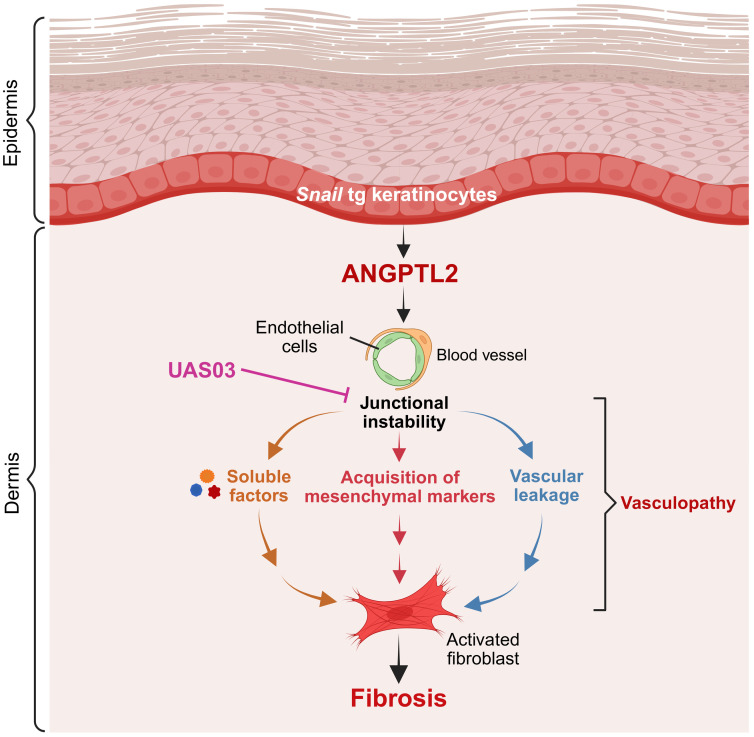
Model demonstrating vasculopathy-mediated fibrosis driven by ANGPTL2 in *Snail*-tg skin is counteracted by UAS03.

Using a mouse model that recapitulates many of the clinical features of SSc (23), we have elucidated the mechanisms underlying vasculopathy that are a hallmark of this and many fibrotic diseases. Our work positions ANGPTL2 secreted from *Snail*-tg keratinocytes as a major driver of the vasculopathy observed in fibrotic skin. ANGPTL2 upregulates fibrogenic molecules in endothelial cells, downregulates canonical endothelial junction protein claudin5, and mediates acquisition of mesenchymal characteristics. The synthetic metabolite UAS03 counteracts the effects of ANGPTL2 in endothelial cells and reduces fibrosis in *Snail*-tg skin in part by reversing the downregulation of claudin5 (Figure 9). These observations are consistent with a previous report that the transgenic overexpression of *Angptl2* in the skin appears to partially phenocopy the *Snail*-tg mouse, in particular the vascular leakage (46).

Previous reports in SSc patient skin indicated that vasculopathy occurs in progressive stages: early (marked by hyperangiogenesis, giant capillaries), active (marked by aberrant angiogenesis and morphological changes), and late (marked by loss of angiogenesis and avascular areas) (53). Our investigations of the status of the vasculature in the dorsal skin of the neonatal to adult (2-month-old) *Snail*-tg mouse indicate that this model represents the early to active stages of vasculopathy seen in SSc patient skin. Markers indicative of the fibroproliferative stage of SSc include pro-angiogenic factors such as VEGF (54). Our previous work demonstrated upregulation of VEGF in *Snail*-tg skin (55). Furthermore, Ki67 staining (Figure 1D) is consistent with proliferation driving higher vascular density (quantified in Figure 1B). Cumulatively, the evidence of the pro-angiogenic signaling further supports our claim that *Snail*-tg skin represents the early stages of the disease at the time points investigated in this study. Although there is an observed increase in vascular density and numbers, the quality of the vasculature is compromised, leading to leakage. This can be compared to cancer angiogenesis wherein there is formation of many new vessels but their quality is poor (56). The loss of vasculature (a feature of the late stage of the disease) might be observed much later in the lifetime of the *Snail*-tg mouse, which we have not included in this study. We have previously reported necrosis in the tail of the *Snail*-tg mouse (23), and this could be representative of the loss of vessels. The majority of SSc patients seek medical treatment at later stages of the disease when destructive vasculopathy is observed. We believe that early and active vasculopathy is faithfully recapitulated in the *Snail*-tg skin in the ages we have included in this study and that this would be a useful platform for identifying biomarkers for detecting early stages of the disease as well as developing therapeutics to combat the disease development before severe damage.

To date, it has not been clearly elucidated whether vasculopathy is a cause or a secondary effect of fibrosis in diseases such as SSc. Our work positions vasculopathy as an integral component driving fibrogenesis rather than a mere symptom of the disease. An outstanding question is how vasculopathy connects to other aspects of the disease pathology, such as inflammation. Platelet activation has recently been hypothesized to be an important link between vasculopathy and inflammation in SSc (57). Platelets contain granules that store various factors that can be involved in SSc vasculopathy, including PDGF and VEGF (58). Intact vessel walls in unaffected vasculature usually prevent any stimuli causing platelet activation; however, activated platelets can release granules containing these factors as well as molecules with proinflammatory activities. Endothelial dysfunction is an early event of SSc pathogenesis that leads to platelet activation (57). Along with ANGPTL2-mediated junctional perturbation, this can potentially be an early inducer of platelet activation, setting in motion the subsequent changes in the pathogenesis of SSc.

An interesting observation in our study is that vascular defects and secretion of ANGPTL2 begin in neonatal stages in *Snail*-tg skin. Previous reports have revealed that vasculopathy precedes other pathological features in SSc patient skin (59), and our data concur with this clinical observation. We thus propose that ANGPTL2 can serve as a possible biomarker for detecting SSc-like pathologies at an early stage before the manifestation of dermal thickening and accumulation of ECM, which are gross indications of fibrosis. Therapeutic modalities targeting ANGPTL2 can also potentially be of relevance in fibrotic diseases beyond SSc. Our analysis of published datasets has revealed upregulation of *Angptl2* in other fibrotic tissues, such as the lung, kidney, and liver (Supplemental Figure 4B). In addition to fibrosis, vascular dysfunction is an integral component in the development of several pathologies, such as inflammatory diseases and cancers (60, 61), which likewise exhibit elevated levels of ANGPTL2 (62).

Together with previous reports, our data suggest that ANGPTL2 does not have a role in homeostasis. ANGPTL2 is not required for normal vascular development, and the loss of ANGPTL2 in mice (46) or zebrafish (63) does not affect the animal’s viability and fertility. Furthermore, we observed no prominent effect on wound closure in the *Angptl2*-KO mouse skin (Supplemental Figure 9A). This suggests that the role of ANGPTL2 is primarily in pathological scenarios. Consistent with this, previous reports have shown ANGPTL2 to have important roles in tumor angiogenesis in various cancers (40, 64, 65). This indicates that a specific inhibitor to ANGPTL2 would likely have less side effects on normal tissue and thus have a high therapeutic potential.

As vasculopathy is a prominent feature of fibrotic diseases, previous therapies targeted factors such as *Edn1* (66) and VEGF (67). Although promising in preclinical studies, they yielded limited success in clinical trials (66, 67). Therefore, it may be beneficial to target upstream mediators of these pathways that would theoretically have a broader effect on multiple downstream pathways. Our work has revealed that UAS03 targets multiple pathways downstream of ANGPTL2, leading to a reduction in vasculopathy-mediated fibrogenesis. The metabolite Urolithin A and its synthetic analog UAS03 have previously been reported to aid in gut epithelial junctional stability and in the reduction of inflammation in a mouse model of inflammatory bowel disease (48). Although previous reports attributed its anti-inflammatory effects to an inhibition of macrophage activity, blocking activated macrophages via clodronate liposomes in our study was not sufficient to reduce the vasculopathy and fibrosis in *Snail*-tg skin (Supplemental Figure 7). Therefore, utilizing UAS03 as a therapy would not only affect inflammation but also other important signaling pathways that drive fibrogenesis. Interestingly, UAS03-treated animals did not exhibit a defect in wound closure, suggesting its effect is limited to pathological scenarios (Supplemental Figure 9B). Therefore, our work provides important evidence that supports using UAS03 as a potential therapeutic approach for vasculopathy-mediated fibrosis development.

## Methods

### Sex as a biological variable.

Mice of both sexes were used for the study, and similar results were observed. Clinical samples from SSc patients were all from female donors.

### Animal studies.

WT (CD1) mice were obtained from The Jackson Laboratory. The K14-*Snail*-tg mice were engineered as described previously (68). The *Angptl2*-KO mouse was developed at the Mouse Genome Engineering Facility at Bangalore Life Sciences Cluster according to a previous report (46). The *Snail*-tg/*Angptl2*-KO mouse was developed by breeding K14-*Snail*-tg and *Angptl2*-KO mice. The *Snail*-tg/*Mindin*-KO and *Snail*-tg/*Pai1*-KO mice were developed as previously reported (23, 24).

### Immunostaining and histology.

Skin tissue was embedded in OCT (Leica) for sectioning. The 20 to 30 μm thick sections, whole skin mounts, or cells on coverslips were fixed using 4% paraformaldehyde and used for staining. H&E staining was performed to observe tissue histology. The primary antibodies and dilutions used for immunofluorescence staining were as follows: PECAM1 (BD Biosciences; catalog 550274; 1:150), Ki67 (Abcam; catalog AB16667; 1:200), claudin5 (Invitrogen; catalog 34-1600; 1:50), VE-Cadherin (BD Biosciences; catalog BD550548; 1:50), ANGPTl2 (R&D Systems; catalog AF1444; 1:50), α-SMA (Sigma; catalog A2547; 1:200), collagen I (Abcam; catalog AB21286; 1:200), β-catenin (Millipore; catalog 05-665; 1:500), and CD11b (DSHB; catalog M1/70.15.11.5.2; 1:200). Alexa Fluor 488– and Alexa Fluor 561–labeled secondary antibodies (Jackson ImmunoResearch) were used at a dilution of 1:300. Alexa Fluor 568 Phalloidin (Invitrogen; catalog A12380) was used to stain actin. Nuclei were marked by DAPI.

### Image collection and analysis.

Imaging was performed with an Olympus IX73 microscope, an Olympus FV3000 confocal microscope, or a Nikon A1R confocal microscope. Images were analyzed on ImageJ (Fiji) software. For morphometric analysis of blood vessels in the skin, vessel density was calculated by counting total vessel profiles divided by area of tissue in millimeters squared; percentage mean vessel area was calculated as, (total vessel area/total tissue area) × 100. Collagen staining was quantified by integrated density measurement of the staining in the dermal region. Dermal thickness was quantified by measuring the area of the dermis in the H&E-stained sections. Quantifications for claudin5, VE-Cadherin, and α-SMA were done by counting double-positive vascular structures for PECAM1 and these markers, respectively. Three fields of interest (FOIs) were analyzed per biological replicate for quantification of images.

### RNA extraction, cDNA synthesis, and qPCR.

Total RNA was extracted from skin biopsies and cell lysates using RNAiso Plus Reagent (Takara; catalog 9109) according to the manufacturer’s protocol. cDNA was prepared using a PrimeScript cDNA synthesis kit (Takara; catalog 2680A) according to the manufacturer’s instructions. qPCR was performed using PowerUp SYBR Green Master Mix (Applied Biosystems, Thermo Fisher Scientific; catalog A25742) with a Bio-Rad CFX384 thermal cycler. TATA-box binding protein (TBP) expression was used for normalization. The primers used are listed in Table 1.

### Evans blue dye injection assay.

Evans blue dye (1% w/v in PBS) (Sigma; E2129) was injected into the retro-orbital sinus of the mice according to body weight (60 mg/kg). The mice were then euthanized, and dorsal skin tissue was harvested. The tissue samples were air-dried and then incubated with formamide at 55°C to extract the Evans blue dye into solution. The absorbance of this solution was measured at 620 nm. Extravasated Evans blue dye (nanograms per milligram of tissue) was calculated using a standard curve.

### FACS-based isolation of cells from mouse skin.

Dorsal skin of either P3 or adult (P60) WT and *Snail*-tg mice was harvested, and all the hair was removed. For P3 mice, the dermis was separated from the skin by incubating in 1 mg/mL dispase for 1 hour at 37°C. For P60 mice, the whole skin was processed. The tissue was digested with 2.5 mg/mL collagenase IV (Gibco) for either 45 minutes or 2 hours at 37°C. The cell suspensions were stained with PECAM1-FITC antibody (MACS Miltenyi Biotech; catalog 130-102-970; 1:50) or CD45-PE antibody (MACS Miltenyi Biotech; catalog 130-102-781; 1:100) at 4°C for 30 minutes. PECAM1^+^, CD45^+^, and lineage-negative cells were sorted from this cell suspension using BD FACS Aria Fusion or Aria III (BD Biosciences). Laser delay and area scaling were done using Sphero beads (BD Biosciences). ACD beads (BD Biosciences) were used for setting drop delay. Unstained samples were used for setting the voltages. The cell population was gated using side scatter area and forward scatter area (FSC-A) plots. A diagonal gate was made to select single cells using FSC-A and forward scatter height plots. The dead cell population was identified by staining with propidium iodide (Sigma; catalog P4864), and a dead cell population of less than 15% of all events and less than 10% of gated cells was considered for proceeding. Single-stained PECAM1 and CD45 samples were then recorded and color compensation performed. Thereafter, costained tubes of samples were used to sort PECAM1^+^, CD45^+^, and lineage-negative cells. For the PECAM1^+^ cells from adult skin, back sorting was performed to ascertain purity of cells (Supplemental Figure 10). The sorted cells were pelleted at 100*g*, and RNA was extracted directly as described previously.

### Cell culture and preparation of conditioned media.

Mouse endothelial cell line SVEC4-10 (ATCC CRL-2181) was purchased from ATCC. SVEC4-10 cells were cultured in DMEM high-glucose media with 10% FBS. For all staining of SVEC4-10 cells, cells were grown on a coverslip coated with 50 μg/mL collagen I solution until they formed an endothelial sheet, and treatments were done subsequently. A SVEC4-10 cell line with constitutive claudin5 overexpression (SVEC4-10-C5) was developed by transfecting pcDNA3.1 plasmid containing the mouse *Cldn5* gene.

Primary mouse keratinocytes were isolated from epidermis of P3 pups and either directly processed for RNA isolation or cultured in low-calcium E-media to maintain an undifferentiated proliferating state, as described previously (69, 70). For collection of conditioned media from keratinocytes for treatment, serum-free media was added to confluent plates of cells, and conditioned media collected after 16 hours.

Primary newborn dermal fibroblasts were isolated from dermis of P3 pups as described earlier (25). Fibroblasts were cultured in DMEM high-glucose media supplemented with 10% FBS, sodium pyruvate, and nonessential amino acids.

The CHO cell line (CHO K1; ATCC CCL-61) was purchased from ATCC. A CHO cell line stably secreting ANGPTL2 was developed by transfecting pcDNA3.1 plasmid containing the mouse *Angptl2* gene. Conditioned media from CHO cells with an empty plasmid was used as control in all the experiments. CHO cells were maintained in DMEM high-glucose media with 10% FBS. For preparation of conditioned media, CHO cells secreting ANGPTL2 and control CHO cells were incubated with media containing 2% FBS for 3 days, and the conditioned media was collected and snap-frozen before storing at –80°C. For all treatments of SVEC4-10 endothelial cells, this conditioned media was used directly to treat for 48 hours (for qPCR analysis) and 72 hours (for collagen contraction assay). Concentration of ANGPTL2 for treatments was 500 ng/mL.

### Ex vivo treatment of skin explants and ears.

WT adult mouse ears or back skin explants were obtained immediately after sacrificing mice by cervical dislocation and placed in PBS with antibiotics and antifungal drugs (PenStrep, Gentamicin, and Fungizone, Thermo Fisher Scientific) for 2 hours. Explants were transferred to cell culture plates (dermis side down) containing CHO cell line stably secreting ANGPTL2 or control CHO cells. After 5 days explants were removed, and staining and RNA extraction were done as described above. For treatments with WT and *Snail*-tg keratinocyte conditioned media, explants were similarly incubated with respective conditioned media prepared as described previously.

### ELISA.

The ELISA for ANGPTL2 (Novus Biologicals; catalog NBP2-68213) was performed on conditioned media (16 hours) from cultured WT and *Snail*-tg mouse keratinocytes according to the manufacturer’s protocols.

### Analysis of published datasets for Angptl2 gene expression.

The normalized expression of *Angptl2* in keloids, SSc, lung with idiopathic pulmonary fibrosis, kidney glomeruli with lupus nephropathy, and liver with fibrosis after transplant was obtained from publicly available microarray data (GSE92566, GSE181549, GSE48149, GSE32591, and GSE145780). The GEO2R tool (NCBI) was used to analyze the expression level difference between 2 groups. An unpaired Welch’s *t* test was used to identify significance level between 2 groups.

### Human samples.

SSc patient samples and appropriate control skin samples were obtained according to the protocol approved by the Institutional Review Board. Skin-punch biopsies were taken from the arms of patients diagnosed with diffuse SSc or nonsystemic sclerosis. Details of patients are listed in Table 2.

### Collagen contraction assay.

The assay was performed as described previously (71). SVEC4-10 or SVEC4-10 cells with claudin5 overexpression were used. Briefly, cells were seeded at a density of 120,000 cells in plugs made of rat tail collagen I (Millipore; 08-115) in a 24-well plate, and various treatments were added. Collagen plugs were stained with crystal violet, and contraction was measured from the gel images after 72 hours. The values are represented as fold change of increase in contraction (1/area of collagen gel). Three technical replicates were used for each condition in each biological replicate.

### Inhibitor studies.

Experiments to inhibit the integrin α_5_β_1_ receptor and β-catenin were performed by treating SVEC4-10 cells with ATN-161 (50 μM) (TOCRIS; catalog 6058/10) and XAV-939 (10 μM) (Selleckchem; catalog S1180), respectively, in the presence of ANGPTL2 for 48 hours (for qPCR analysis) or 72 hours (for collagen contraction assay).

### In vitro permeability assay.

The FITC-dextran permeability assay was performed according to previous reports (72, 73). Per insert, 2 × 10^5^ SVEC4-10 cells were seeded in a 24-well Transwell plate and incubated for 72 hours in a 37°C/5% CO_2_ tissue culture incubator to form a confluent monolayer. The growth medium was carefully removed from the inserts so as not to disturb the monolayer, and the inserts were placed in fresh plate wells. Media containing control, ANGPTL2, or UAS03 was added to both inserts (200 μL) and wells (500 μL), and cells were treated for 48 hours in the incubator. The treatment media was removed carefully from the inserts and washed with PBS. The inserts were again placed into fresh well plates containing 500 μL PBS. A working solution of FITC-dextran (Merck; catalog 90718) in PBS (1 mg/mL) was prepared, and 150 μL was added to the inserts. The plate was incubated, protected from light, for 20 minutes at room temperature. Permeation was stopped by removing the inserts from the wells. The media in the wells of the receiver tray (now containing FITC-dextran that crossed the monolayer) was thoroughly mixed. One hundred microliters of the media from each well of the receiver tray was removed and transferred to wells of a black 96-well opaque plate for fluorescence measurement using a fluorescence plate reader with filters appropriate for 485 and 535 nm excitation and emission, respectively.

### UAS03 treatments.

For in vivo experiments, UAS03 (20 mg/kg body weight) was injected interperitoneally once a week starting from P3 to P60 when mice were sacrificed. For all in vitro experiments with SVEC4-10 cells, 50 μM UAS03 in DMSO formulation was used.

### Clodronate treatments.

Clodronate liposomes to deplete macrophages were injected (1 mg) in mice once a week starting from P3 to P60 when mice were sacrificed.

### RNA-Seq library preparation and data analysis.

RNA was isolated from control (DMSO) or 50 μM UAS03–treated (48 hours) SVEC4-10 cells (3 biological replicates each) as previously described. Integrity of the RNA samples was assessed using the Agilent TapeStation with High Sensitivity RNA ScreenTape. Samples with a RNA integrity number greater than 5 were selected for library preparation. mRNA was enriched through oligo-dT bead selection using the Dynabeads mRNA Purification Kit (Invitrogen). The enriched mRNA was then used for downstream library preparation with the MGIEasy RNA Library Prep Kit (MGI; catalog 1000005276). Libraries were PCR amplified and circularized using the MGIEasy Circularization Module (MGI; catalog 1000005260). These circularized libraries were transformed into DNA Nanoballs and sequenced on the DNBSEQ-G400RS platform using FCL PE100 flow cell chemistry (MGI; catalog 1000016949 and 1000016985). Approximately 30–50 million reads were obtained from both control and UAS03-treated samples. The analysis was performed in house following an RNA-Seq analysis pipeline previously established in the field. The FastQC tool (https://www.bioinformatics.babra ham.ac.uk/projects/fastqc/) was used for assessing the quality of Fastq files. Abundance values were generated with kallisto (74) by aligning them against mouse cDNA sequences (Mus_musculus.GRCm39.cdna.all.fa.gz; https://ftp.ensembl.org/pub/release-113/fasta/mus_musculus/cdna/). The kallisto output was imported in R using tximport, and the ensDB.MMusculus.v79 database was used for annotation of transcripts. The counts from abundance were calculated using the length-scaled transcripts per million (TPM) method. Genes that had a count value greater than 0 in 3 or more samples were selected and normalized using the calcNormFactors function using trimmed mean of M-values (TMM) method. Limma and edgeR packages were used to create a linear fit and discover the differentially expressed genes using the eBayes function. gplots and ggplot were used for creating the heatmap and volcano plots. Modules containing lists of upregulated genes (*q* value < 0.05 and fold change > 1.5) and downregulated genes (*q* value < 0.05 and fold change > –1.5) were used as input for gProfiler2 (75) for GO term enrichment analysis.

### Wounding studies.

Twelve-week-old (a) WT mice injected with control or UAS03 or (b) WT and *Angptl2*-KO mice were wounded with 5 mm punch biopsies, and wounded skin was imaged until complete wound closure. Wound areas were calculated as a percentage of the open wound.

### Statistics.

Comparison of 2 groups was done using either unpaired Welch’s *t* test (for in vivo experiments) or paired 1-tailed Student’s *t* test (for in vitro experiments with cells). Comparison of multiple groups was done using 1-way ANOVA followed by Tukey’s post hoc analysis. Data are shown as mean ± SEM. GraphPad Prism 5.02 was used for all statistical analyses. *P* values of less than 0.05 were considered significant.

### Study approval.

Animal work in the Jamora laboratory was approved by the inStem Institutional Animal Ethics Committee (INS-IAE-2019/06[R1]). Acquisition and processing of the human tissue were conducted according to the protocol approved by the Institutional Review Board of the Sanjay Gandhi Postgraduate Institute of Medical Sciences. Informed consent was acquired from all patients for skin sample collection and experimentation. All experimental work was done with approval of the Institutional Biosafety Committee at inStem [inStem/G-141(3)/2012] and Shiv Nadar Institution of Eminence (95/2024).

### Data availability.

RNA-Seq data for UAS03-treated SVEC4-10 cells used in this study are deposited in NCBI SRA database BioProject (accession ID PRJNA1207732; http://www.ncbi.nlm.nih.gov/bioproject/1207732). Values for all data points in graphs are reported in the Supporting Data Values file.

## Author contributions

DS and CJ conceptualized and designed experiments, evaluated and interpreted data, and wrote the manuscript. DS, RKA, ST, NPS, SK, BD, AD, A Hegde, A Hiwale, VR, and SS performed experiments. DS, RKA, and ST acquired and analyzed data. SK performed bioinformatics analysis and acquired and analyzed data. LMR, NN, NS, and VA provided resources (SSc samples). PKV provided resources (UAS03) and guidance for experimental design. CJ provided guidance and acquired funding.

## Supplementary Material

Supplemental data

Supplemental table 1

Supplemental table 2

Supporting data values

## Figures and Tables

**Table 2:**
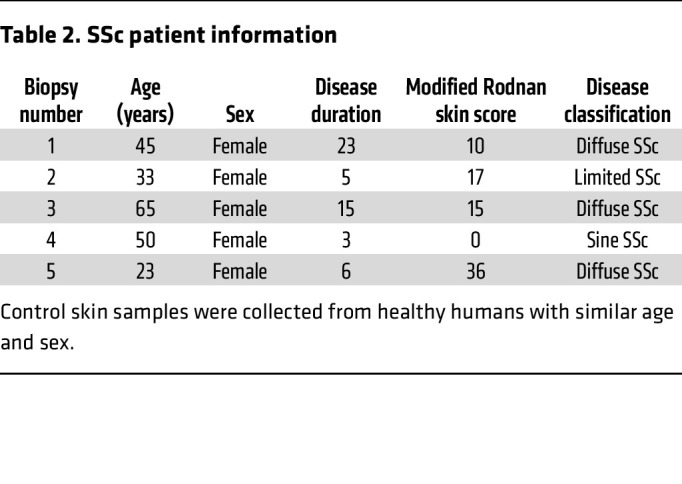
SSc patient information

**Table 1:**
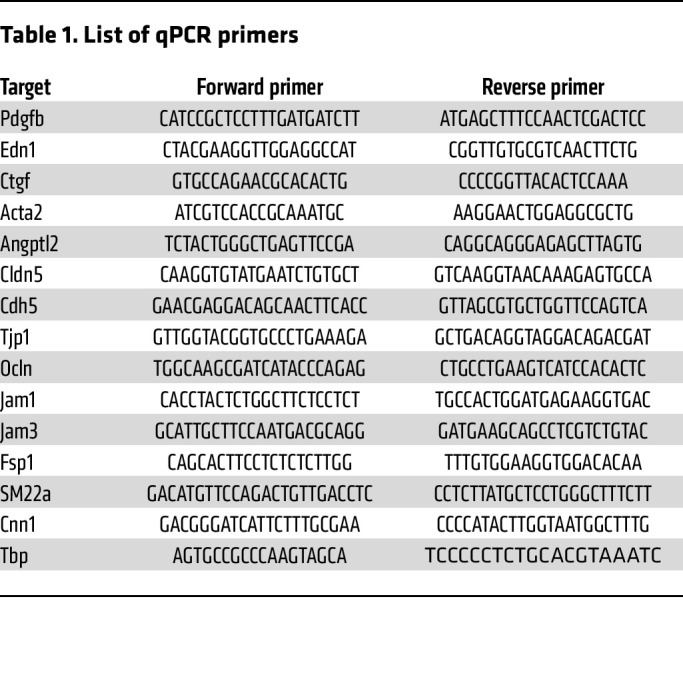
List of qPCR primers

## Version 1 06/20/2025

In-Press Preview

## Version 2 08/01/2025

Electronic publication
