# Supplementary material for: Angiopoietin-like protein 2 mediates vasculopathy-driven fibrogenesis in a mouse model of systemic sclerosis
Source: J Clin Invest. 2025 Jun 20;135(15):e177123. doi: 10.1172/JCI177123 (PMC12321390; doi:10.1172/JCI177123)
Supplement: Supplemental data [file jci-135-177123-s250.pdf]

Supplementary data

Supplementary Figure 1

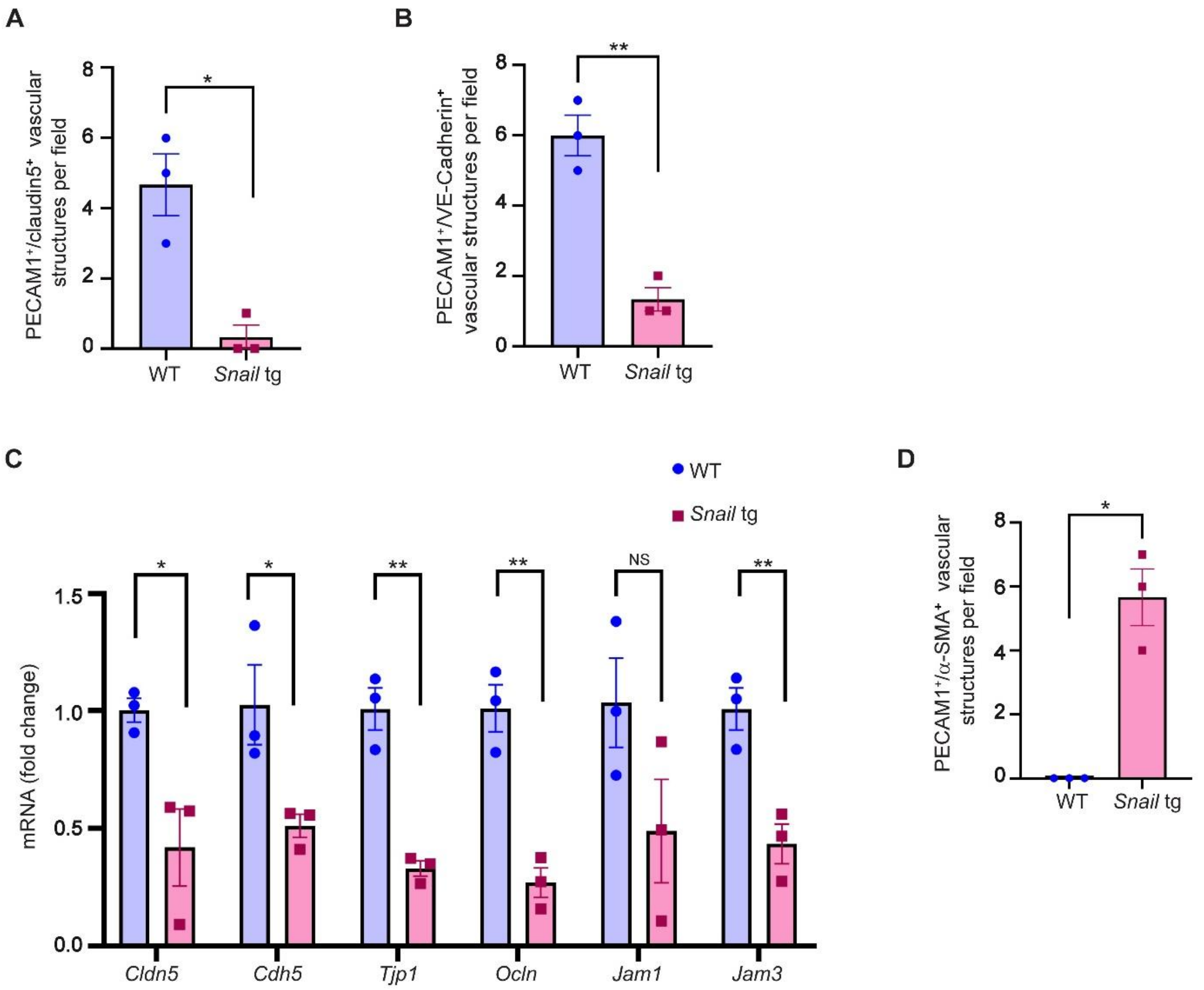

Supplementary Figure 1: Adult *Snail* tg skin recapitulates the junctional perturbation and endoMT observed in SSc. Quantification of (A) PECAM1$^+$/claudin5$^+$ and (B) PECAM1$^+$/VE-Cadherin$^+$ vascular structures in wild type (WT) and *Snail* tg mice skin. (C) qPCR analysis of gene expression of junction proteins (*Cldn5*, *Cdh5*, *Tjp1*, *Ocln*, *Jam1* and *Jam3*) in isolated endothelial cells from WT and *Snail* tg mice skin. (D) Quantification of PECAM1$^+$/α-SMA$^+$ vascular structures in WT and *Snail* tg mice skin. Data are shown as mean ± SEM, p-values were calculated using unpaired Welch's t test. * $p < 0.05$, ** $p < 0.01$, NS > 0.05. All experiments are n=3 biological replicates. 3 FOI were analysed per biological replicate for quantification of images.

Supplementary Figure 2

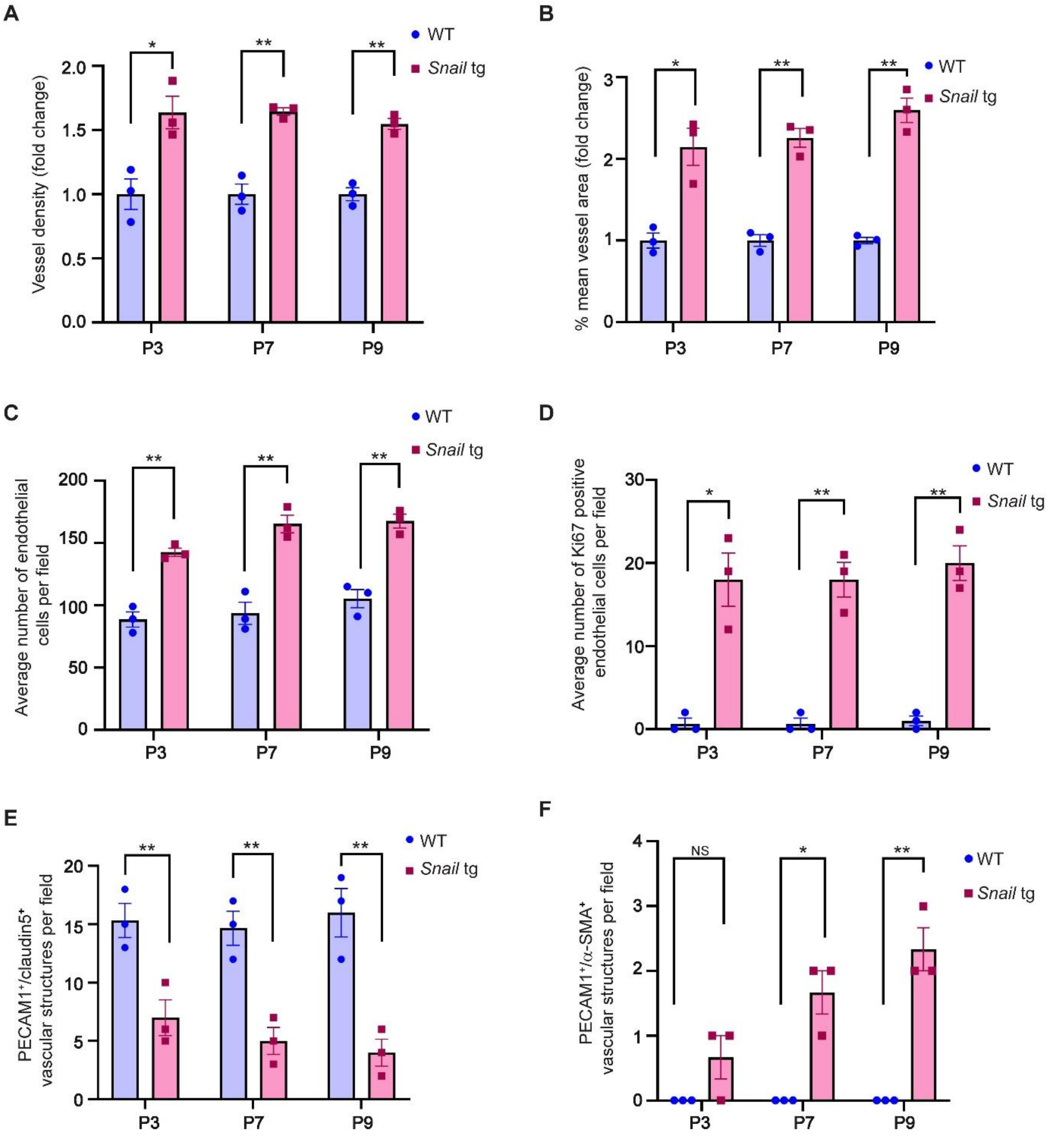

Supplementary Figure 2: Developmental analysis of vascular morphometrics and junctional perturbation in *Snail* tg mouse skin. Quantification of (A) vessel density, (B) mean vessel area, (C) number of endothelial cells, (D) PECAM-1$^+$/Ki67$^+$ cells, (E) PECAM1$^+$/claudin5$^+$ vascular structures and (F) PECAM1$^+$/α-SMA$^+$ vascular structures in wild type (WT) and *Snail* tg mice skin at postnatal day 3, 7, and 9. Data are shown as mean ± SEM, p-values were calculated using unpaired Welch's t test. * $p < 0.05$, ** $p < 0.01$, NS > 0.05. All experiments are n=3 biological replicates. 3 FOI were analysed per biological replicate for quantification of images.

Supplementary Figure 3

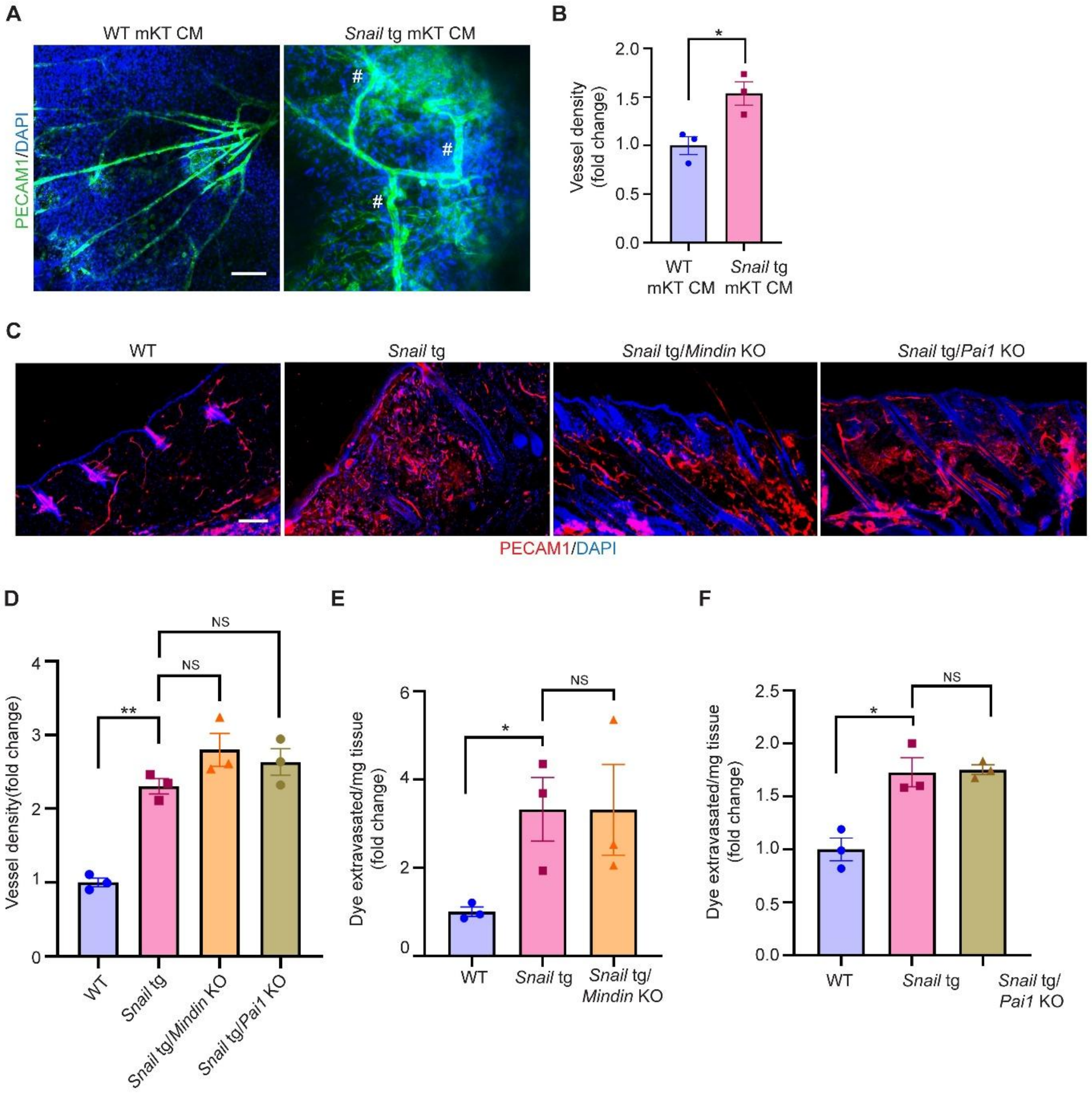

Supplementary Figure 3: Mindin and PAI1 do not mediate the vasculopathy in the *Snail* tg skin. (A) PECAM1 (green) in skin explants treated with wildtype (WT) or *Snail* tg mouse keratinocyte (mKT) conditioned media. Dilated regions are marked by #. (B) Quantification of vessel density in skin explants treated with WT or *Snail* tg mKT conditioned media. (C) PECAM1 (red) in WT, *Snail* tg, *Snail* tg/*Mindin* KO and *Snail* tg/*Pai1* KO skin at P60. (D) Quantification of vessel density in WT, *Snail* tg, *Snail* tg/*Mindin* KO and *Snail* tg/*Pai1* KO skin at P60. (E), (F) Quantification of Evan's blue dye leakage assay. Nuclei are marked in blue. Scale bar: 50 µm (A) and 100 µm (C). Data are shown as mean ± SEM, p-values were calculated using unpaired Welch's t test (B) and one-way ANOVA followed by Tukey's post hoc analysis (D-F). * $p < 0.05$, ** $p < 0.01$, NS > 0.05. All experiments are n=3 biological replicates. 3 FOI were analysed per biological replicate for quantification of images.

Supplementary Figure 4

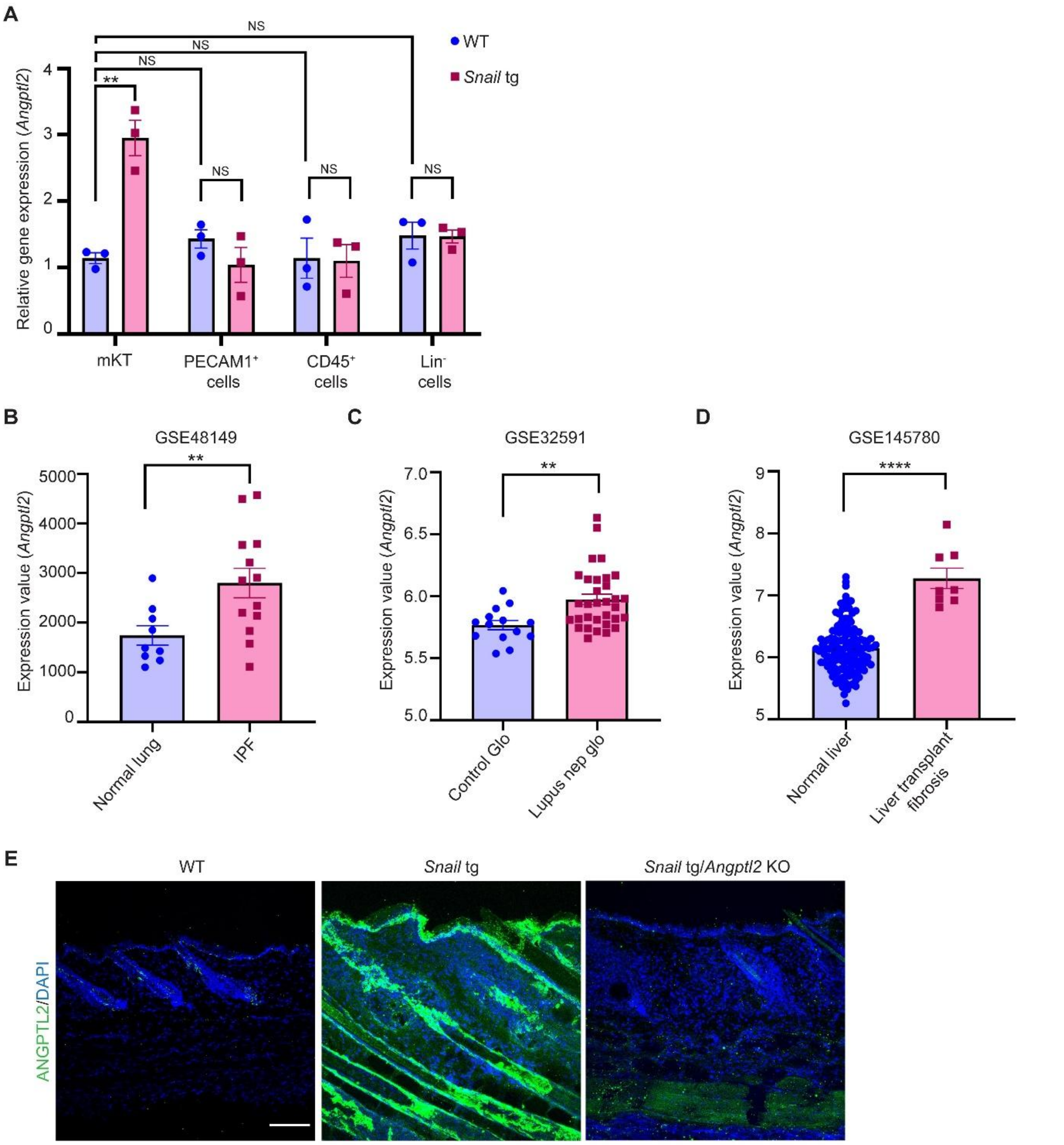

Supplementary Figure 4: ANGPTL2 is expressed by *Snail* tg keratinocytes and upregulated in human fibrotic diseases. (A) qPCR analysis of relative gene expression of *Angptl2* (normalized to housekeeping gene) in isolated keratinocytes (mKT), PECAM1$^+$ cells (endothelial cells), CD45$^+$ cells (primarily immune cells) and lineage negative cells (lin$^-$, primarily fibroblasts) from wild type (WT) and *Snail* tg mice skin. Expression of *Angptl2* in (B) lung with idiopathic pulmonary fibrosis (n=13 samples) compared to normal lung tissue (n=9 samples) (from GSE48149), (C) kidney glomeruli with lupus nephropathy (n=32 samples) compared to control glomeruli (n=14 samples) (from GSE32591), (D) Liver with fibrosis post-transplant (n=8 samples) compared to normal liver tissue (n=129 samples) (from GSE145780). The expression values are fetched from the GEO2R algorithm's output. (E) Immunofluorescence staining for ANGPTL2 (green) in WT, *Snail* tg and *Snail* tg/*Angptl2* KO skin. Nuclei are marked in blue. Scale bar: 100 µm. n=3 biological replicates except (B-D).

Data are shown as mean ± SEM, p-values were calculated using unpaired Welch's t test. ** p < 0.001, **** p < 0.0001, NS > 0.05.

Supplementary Figure 5

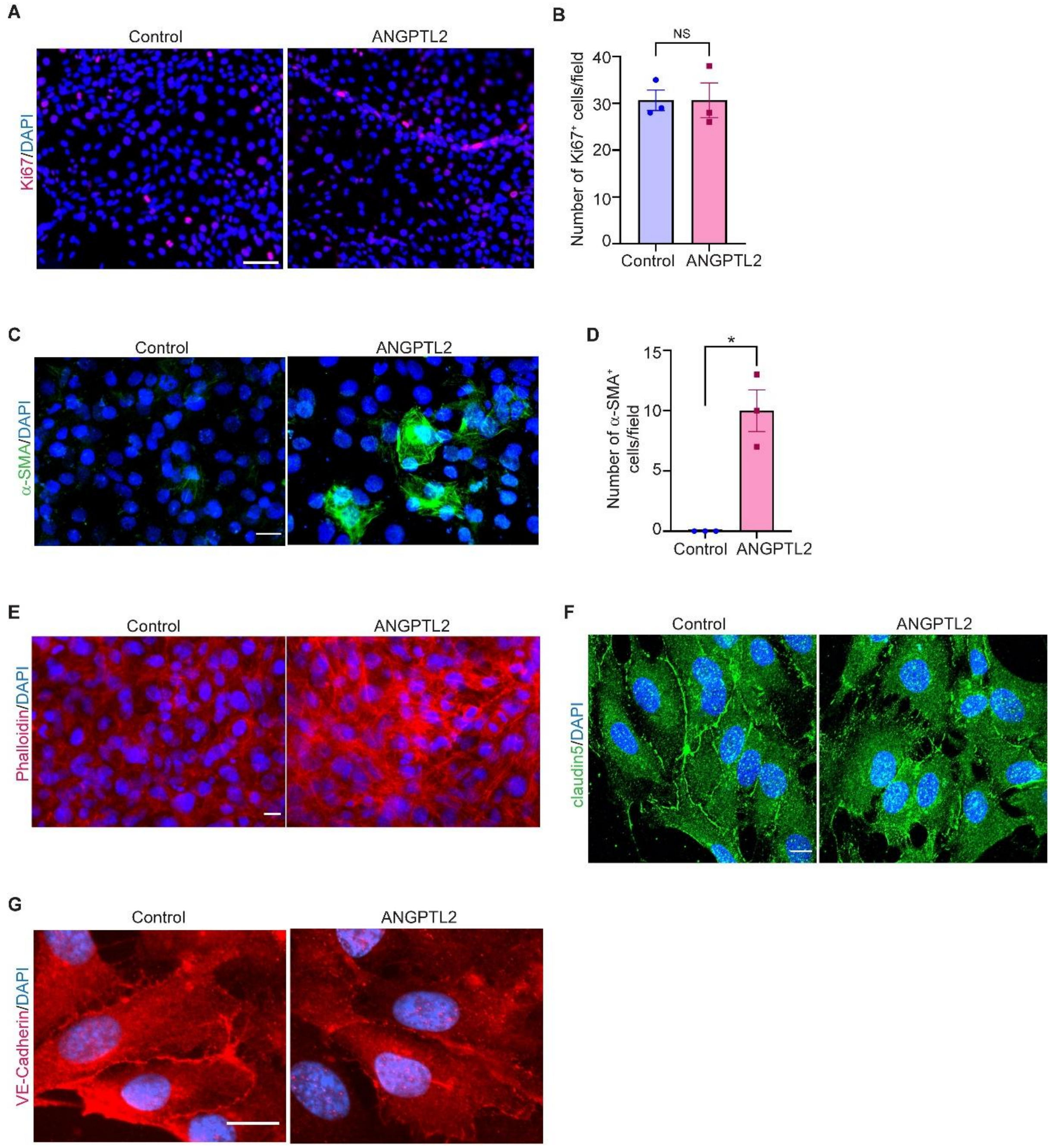

Supplementary Figure 5: Effect of ANGPTL2 on endothelial cells. Immunofluorescent staining and quantification for (A, B) Ki67 (red) and (C, D) α-SMA (green) in control and ANGPTL2 treated SVEC4-10 cells. Immunofluorescent staining for (E) actin marked by Phalloidin (red), (F) claudin5 (green) and (G) VE-Cadherin (red) in control and ANGPTL2 treated SVEC4-10 cells. Nuclei are marked in blue. Scale bar: 50 μm (A, C) and 10 μm (E-G). Data are shown as mean ± SEM, p-values were calculated using paired Student's *t* test. * $p < 0.05$, NS > 0.05. All experiments are n=3 biological replicates. 3 FOI were analysed per biological replicate for quantification of images.

Supplementary Figure 6

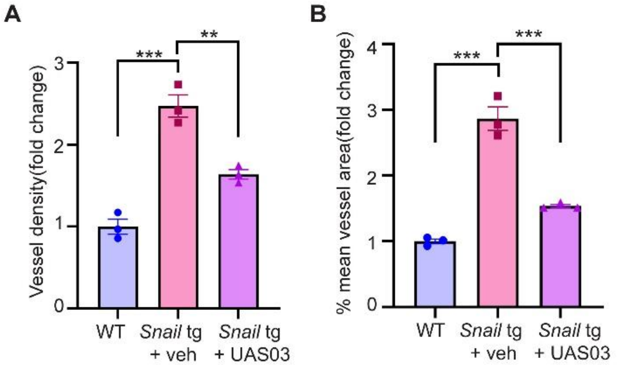

Supplementary Figure 6: UAS03 reduces vasculopathy. Quantification of (A) vessel density and (B) mean vessel area at P60 in wild type (WT), *Snail* tg + vehicle control (veh) and *Snail* tg + UAS03 injected mice. Data are shown as mean ± SEM, p-values were calculated using one-way ANOVA followed by Tukey's post hoc analysis for multiple group comparisons. *** $p < 0.001$, ** $p < 0.01$. All experiments are n=3 biological replicates. 3 FOI were analysed per biological replicate for quantification of images.

Supplementary Figure 7

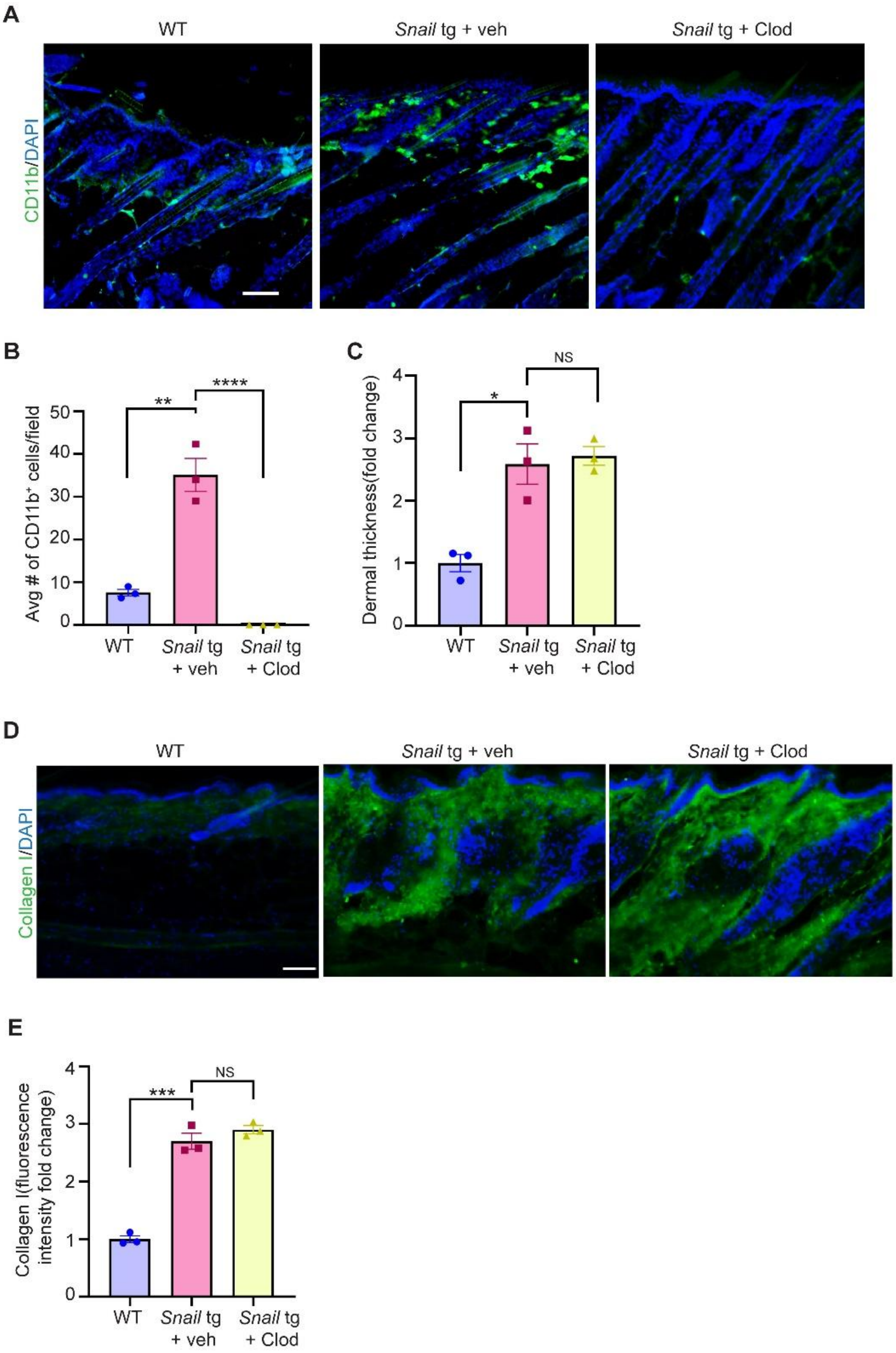

Supplementary Figure 7: Depleting macrophages does not reduce fibrosis. Staining (A) and (B) quantification of macrophages marked by CD11b (green) at P60 in wild type (WT), *Snail* tg + vehicle control (veh) and *Snail* tg + Clodronate (Clod) injected mice. Nuclei are marked in blue. (C) Quantification of dermal thickness at P60 in WT, *Snail* tg vehicle control and *Snail* tg Clodronate injected mice. Staining (D) and quantification (E) of Collagen I at P60 in WT, *Snail* tg + vehicle control and *Snail* tg + Clodronate injected mice. Scale bar: 50 µm. Data are shown as mean ± SEM, p-values were calculated using one-way ANOVA followed by Tukey's post hoc analysis for multiple group comparisons. * $p < 0.05$, ** $p < 0.01$, *** p <

0.001, **** $p < 0.0001$, NS > 0.05. All experiments are n=3 biological replicates. 3 FOI were analysed per biological replicate for quantification of images.

Supplementary Figure 8

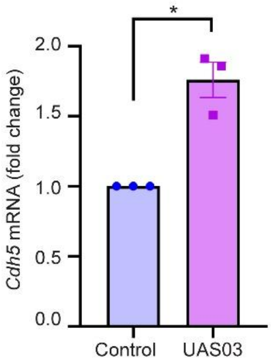

Supplementary Figure 8: UAS03 upregulates endothelial adherens junction component VE-Cadherin. qPCR analysis of mRNA levels of *Cdh5* in SVEC4-10 cells treated with Control or 50 μM UAS03. Data are shown as mean ± SEM, p-values were calculated using paired Student's *t* test. * $p < 0.05$. n=3 biological replicates.

Supplementary Figure 9

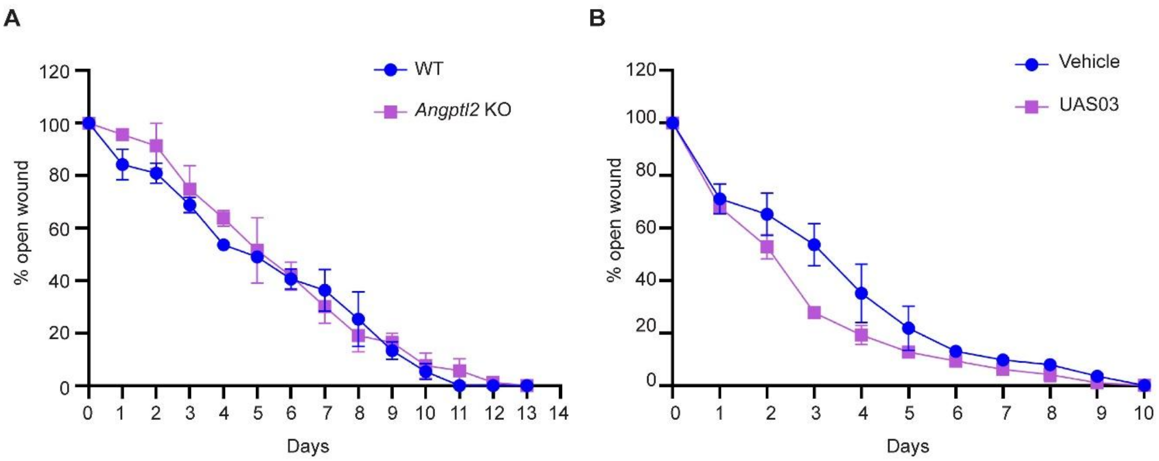

Supplementary Figure 9: Wound closure kinetics in (A) *Angptl2* KO and (B) UAS03 treated mice compared to wildtype counterparts respectively. All experiments are n=3 biological replicates.

Supplementary Figure 10

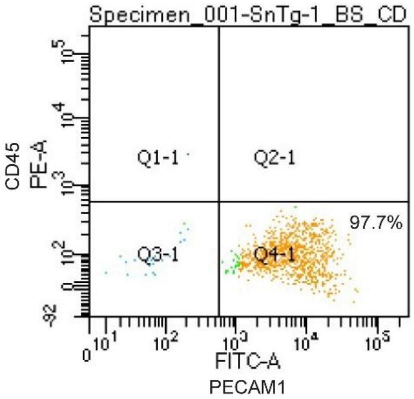

Supplementary Figure 10: Purity of isolated endothelial cells from mouse skin. Plot showing purity of sorting with PECAM1$^+$ endothelial cell population previously sorted from *Snail* tg mouse skin using FACS. >95% cells isolated in a sample were pure PECAM1$^+$ population.

Supplementary Table 1: Differentially expressed genes in UAS03-treated SVEC4-10 cells compared to control-treated cells (available as a separate Excel file).

Supplementary Table 2: GO term enrichment analysis via gProfiler2 using upregulated and downregulated genes respectively from UAS03-treated SVEC4-10 cells compared to control-treated cells (available as a separate Excel file).
